# Sulfated glycosaminoglycans inhibit transglutaminase 2 by stabilizing its closed conformation

**DOI:** 10.1038/s41598-022-17113-2

**Published:** 2022-08-03

**Authors:** Claudia Damaris Müller, Gloria Ruiz-Gómez, Sophie Cazzonelli, Stephanie Möller, Robert Wodtke, Reik Löser, Joanna Freyse, Jan-Niklas Dürig, Jörg Rademann, Ute Hempel, M. Teresa Pisabarro, Sarah Vogel

**Affiliations:** 1 Institute of Physiological Chemistry, Medical Faculty Carl Gustav Carus, Technische Universität Dresden, Fetscherstraße 74, 01307 Dresden, Germany; 2 Structural Bioinformatics, BIOTEC, Technische Universität Dresden, Tatzberg 47-51, 01307 Dresden, Germany; 3 Biomaterials Department, INNOVENT e.V., Prüssingstraße 27 B, 07745 Jena, Germany; 4 Helmholtz-Zentrum Dresden-Rossendorf, Institute of Radiopharmaceutical Cancer Research, Bautzner Landstrasse 400, 01328 Dresden, Germany; 5 Institute of Pharmacy, Freie Universität Berlin, Königin-Luise-Straße 2/4, 14195 Berlin, Germany

**Keywords:** Biochemistry, Chemical biology, Computational biology and bioinformatics, Structural biology

## Abstract

Transglutaminases (TGs) catalyze the covalent crosslinking of proteins via isopeptide bonds. The most prominent isoform, TG2, is associated with physiological processes such as extracellular matrix (ECM) stabilization and plays a crucial role in the pathogenesis of e.g. fibrotic diseases, cancer and celiac disease. Therefore, TG2 represents a pharmacological target of increasing relevance. The glycosaminoglycans (GAG) heparin (HE) and heparan sulfate (HS) constitute high-affinity interaction partners of TG2 in the ECM. Chemically modified GAG are promising molecules for pharmacological applications as their composition and chemical functionalization may be used to tackle the function of ECM molecular systems, which has been recently described for hyaluronan (HA) and chondroitin sulfate (CS). Herein, we investigate the recognition of GAG derivatives by TG2 using an enzyme-crosslinking activity assay in combination with in silico molecular modeling and docking techniques. The study reveals that GAG represent potent inhibitors of TG2 crosslinking activity and offers atom-detailed mechanistic insights.

## Introduction

Transglutaminases (TGs) are multifunctional enzymes which covalently link proteins via intra- and intermolecular isopeptide bonds^1,2^. The respective substrate pair consists of a peptide-bound glutamine residue and an amine-donor e.g. the *N*^ε^-amino group of a peptide-bound lysine (also low-molecular weight amines) forming ε-(γ-glutamyl)-lysine isopeptide bonds. Such covalent crosslinks are stable and resistant to enzymatic, chemical and mechanical cleavage^1,3^. Besides, TGs can hydrolyse glutamine residues to glutamate and exhibit also isopeptidase activity^4,5^.

The human genome encodes eight TG isoenzymes (TG1-7 and factor XIII)^2^, whose activities are associated with different physiological processes such as extracellular matrix (ECM) stabilization, wound healing and inflammation, and also with serious diseases such as cancer, atherosclerosis and celiac disease^6^. Thus, TGs constitute interesting targets for pharmacological applications for the above-mentioned diseases^7,8^*.*

Among the eight known isoenzymes, TG2 is the most prominent isoform being expressed in almost all cell types in the human body. TG2 is a 78 kDa protein consisting of 687 amino acids organized in four structural domains: β-sandwich (residues 1–139), α/β-transamidase (147–460), β-barrel 1 (472–583) and β-barrel 2 (591–687)^9^. The β-sandwich domain contains interaction sites for the tumor suppressor protein p53 (residues 1–139) and fibronectin (residues 88–106). The α/β-transamidase domain includes the catalytic triad (residues C277, H335, D358) and five calcium (Ca^2+^) binding sites (S1 (_226_GMVNCNDD_233_), S2A/B (_396_EVNADV_401_, _447_EGSSEERE_454_), S3A/B (_306_DQNSNLL_312_, _328_SEM_330_), S4 (_151_DSEEERQE_158_) and S5 (_433_RDERED_438_)^10^). Both β-barrel domains provide GTP/GDP binding sites (residues 476–478 and 538–580). It has been reported that interacting cofactors, e.g., GTP or Ca^2+^, are able to alter the spatial arrangement of the four TG2 domains^11^. The closed TG2 conformation is catalytically inactive and stabilized by GTP/GDP acting as a kind of reversible inhibitor bringing the α/β-transamidase domain in close proximity to β-barrel domains 1 and 2^9,11^. On the other hand, the stretched open TG2 conformation is induced and stabilized in the presence of Ca^2+^ through the displacement of the β-barrel domains 1 and 2^11,12^. As reported, six Ca^2+^ are bound to TG2 in five binding sites^10,13^. This open TG2 conformation is associated with the crosslinking activity of the enzyme^14^.

TG2 is located intra- as well as extracellularly^15,16^. In physiological conditions, intracellular-acting TG2 will be mostly inactive because of high GTP and low Ca^2+^ levels in the cell. There, TG2 interacts with nuclear, cytosolic and membrane receptors. For example, intracellular TG2 has been shown to prevent apoptosis non-reliant on its transamidase activity, but largely depending on its GTP-binding capacity^17^. Although the situation in the extracellular space concerning GTP and Ca^2+^ levels is opposite to the intracellular compartment, TG2 might also be mainly inactive there under physiological conditions^11,18^ due to redox regulation, which can lead to the formation of a vicinal disulfide bond between Cys370 and Cys371^19^. As described by Jin et al.^20^, the reduction of this disulfide bond results in an active extracellular TG2.

Irrespective of its activation state, extracellular TG2 has been linked to i.a. cell adhesion, ECM stabilization and maturation, proliferation and cell motility^16,21^. Active TG2 crosslinks many proteins (e.g. fibronectin) in order to form stable well-assembled ECM networks^22,23^*.* Furthermore, TG2 has been reported as a binding partner of the glycosaminoglycans (GAG) heparan sulfate (HS) and heparin (HE)^23–26^.

GAG are linear polysaccharides consisting of disaccharide units built from alternating *N*-acetylated and/or *O*-sulfated uronic acids and glycosamines^27,28^. Based on their chemical properties, GAG can be classified into non-sulfated, e.g. hyaluronan (HA), and sulfated such as HE, HS and chondroitin sulfate (CS). GAG are important in many cellular processes as they interact and, thereby, modulate the function of extracellular proteins including cytokines and structural ECM proteins like fibronectin^29,30^. In the past, naturally occurring GAG have been chemically modified and applied in biomaterial research^31–34^. Since then, many effects of chemically sulfated GAG derivatives on cells and their ECM have been reported. Exemplarily, sulfated HA derivatives promote osteoclast cell adhesion but inhibit their resorption^35^ and alter fibronectin expression, conformation and matrix assembly^36^.

Considering TG2 in closed conformation, numerous HS/HE binding sites including the binding motif XBBXB (B is either R or K, X is a hydrophobic amino acid)^24^ have been previously described (Supplementary Table S2)^23,25,26^. These HS/HE binding sites are conserved within different species and localized within the N-terminal β-sandwich, the α/β-transamidase and the C-terminal β-barrel 2 domains. Previous studies have described contrary effects of HE on TG2 activity including a slight inhibitory potential^26,37^ versus no effect^38^. In particular, Wang et al*.* demonstrated reduced incorporation of FITC-cadaverine in the presence of HE within a wound healing assay detecting in situ TG2 activity by fluorescence microscopy^26^. Gambetti et al*.* also observed a slight inhibition of TG2 activity as well as protection of TG2 by HE against proteolytic degradation and thermal denaturation, which indicates the stabilization of TG2 in the closed conformation by this GAG^37^. In contrast, Scarpellini et al*.* did not observe an inhibitory effect of HE towards TG2 as assayed by the incorporation of biotinylated cadaverine into fibronectin, although the sequence of adding Ca^2+^ and HE was not disclosed^38^. Taken together, binding of HE to TG2 in closed conformation is evidenced, but the influence of this interaction on the enzyme’s transamidase activity is rather unclear. Furthermore, Schmidt et al.^39^ previously demonstrated that a low-sulfated HA (SH1) derivative influenced composition and remodeling of the ECM of human bone marrow stromal cells and increased TG2 protein levels. Inspired by this, the present study investigates the impact of naturally occurring polymeric and synthetically modified polymeric and oligomeric GAG derivatives on TG2 enzymatic activity in order to clarify previously reported contradictory findings and to gain insight into the molecular mechanisms that underly this interaction.

## Results

TG2 enzymatic activity was investigated in the presence of a series of polymeric and oligomeric GAG derivatives (Supplementary Fig. S1 and Table 3). Details of the experimental settings used are shown in Fig. 5.

### TG2 activity is inhibited by polymeric sulfated GAG

**Figure 1:**
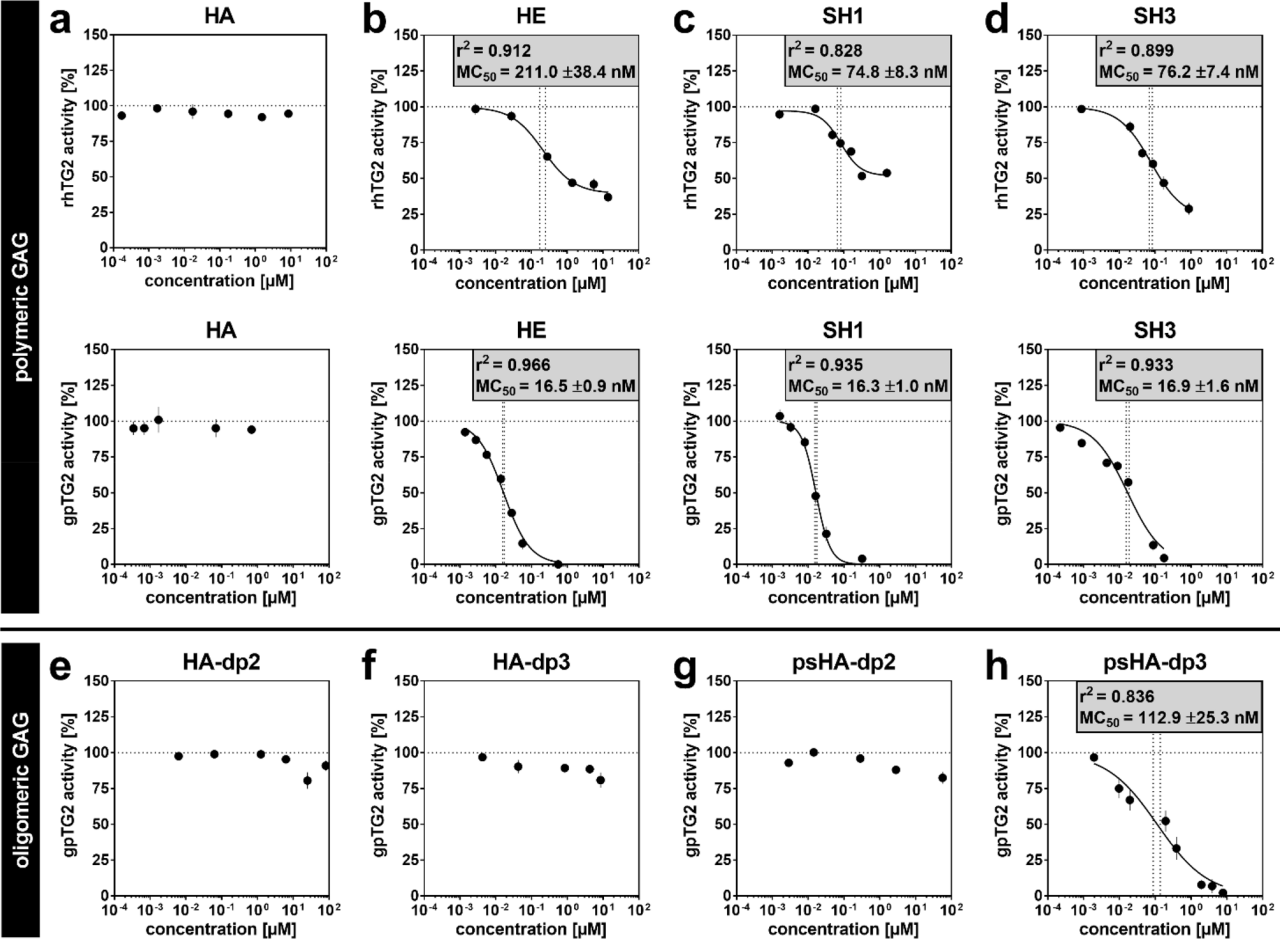
Influence of GAG derivatives on TG2 enzymatic crosslinking activity. rhTG2 (top) and gpTG2 (middle) were pre-incubated with polymeric GAG derivatives (**a**–**d**) and applied to TG2 activity assay as described: (**a**) HA, (**b**) HE, (**c**) SH1 and (**d**) SH3. Furthermore gpTG2 (bottom; **e**–**h**) was incubated with HA oligosaccharides and applied to TG2 activity assay as described: (**e**) HA-dp2, (**f**) HA-dp3, (**g**) psHA-dp2 and (**h**) psHA-dp3. Positive control (i.e. TG2 activity without any treatment) was set to 100%. Values are shown as mean ± SEM; n = 3. MC_50_ values were calculated according to non-linear fit (black line). Vertical dotted lines indicate the range of MC_50_ ± SD.

The influence of several polymeric GAG derivatives on rhTG2 and gpTG2 crosslinking activity was investigated according to experimental setting I (Fig. 5). Complete inhibition to zero activity was not achieved in several cases. This result is unexpected and remarkable, since complete inhibition without residual activity at infinite inhibitor concentration is commonly observed, e.g., as published for the herein used established TG2 inhibitor **7b**^40^. Therefore, MC_50_ values (values of inhibitor/modifier concentration at which the effect is half as strong as the limiting value for the effect at saturating concentration^41^), were calculated by non-linear regression. For a more complete description, the remaining TG2 activities at saturating GAG concentration v_[M]→∞_ are additionally stated in Table 1, which in combination with the MC_50_ values characterize the inhibitory efficiency. In the GAG concentration range examined, [M] = 0.1 nM–10 µM, non-sulfated HA did not have any significant effect on neither rhTG2 (Fig. 1a, top) nor gpTG2 (Fig. 1a, middle). Sulfated GAG derivatives decreased transamidase activity of both homologues in a concentration-dependent manner (Fig. 1b–d). For HE, the MC_50_ value was 211.0 ± 38.4 nM for rhTG2 and 16.5 ± 0.9 nM for gpTG2. However, for rhTG2 residual enzymatic activity v_[M]→∞_ at a presumed infinite HE concentration was still about 40% (Fig. 1b, Table 1). Low-sulfated HA derivative SH1 decreased TG2 enzyme activity with an MC_50_ of 74.8 ± 8.3 nM (rhTG2) and 16.3 ± 1.0 nM (gpTG2) (Fig. 1c). Residual enzymatic rhTG2 activity v_[M]→∞_ was determined to be around 52%. For high-sulfated HA derivative SH3, the obtained values were similar to those of SH1: MC_50_ of 76.2 ± 7.4 nM for rhTG2, and MC_50_ 16.9 ± 1.6 nM for gpTG2 (Fig. 1d). Again, v_[M]→∞_ of rhTG2 was estimated to be about 23%. The inhibitory behavior of medium-sulfated HA derivative SH2 and of CS derivatives with increasing degree of sulfation (D_S_; CS1, CS2 and CS3) was assessed toward gpTG2 (Supplementary Fig. S2). All of these derivatives had comparable MC_50_ values in nM range with negligible enzymatic activity at the highest concentrations applied. Comparing the MC_50_ values and the inhibitory efficiency of all sulfated GAG derivatives for gpTG2, no influence of D_S_ can be stated. For rhTG2, however, the inhibitory efficiency seems to be D_S_-dependent (residual activity values: SH1 > HE > SH3) (Tables 1 and 3). The corresponding MC_50_ values of SH1 and SH3 represent one third of the one obtained for HE.

**Table 1:** MC_50_ values for gpTG2 and rhTG2 of the investigated polymeric and oligomeric GAG derivatives and the irreversible inhibitors. – no inhibition, *n.d.* not determined.

	D_S_	gpTG2		rhTG2	
		MC_50_ ± SD (nM)	v_[M]→∞_ (%)	MC_50_ ± SD (nM)	v_[M]→∞_ (%)
**Polymeric GAG**					
HA	–	–	–	–	–
HE	2.2	16.5 ± 0.9	0.0	211 ± 38.4	39.4
SH1	1.2	16.3 ± 1.0	0.0	74.8 ± 8.3	51.8
SH2	1.9	10.9 ± 1.9	0.0	n.d.	n.d.
SH3	3.2	16.9 ± 1.6	0.0	76.2 ± 7.4	22.7
CS1	0.8	41.0 ± 4.1	13.4	n.d.	n.d.
CS2	1.8	9.4 ± 1.1	0.0	n.d.	n.d.
CS3	2.8	11.9 ± 1.3	0.0	n.d.	n.d.
**Oligomeric GAG**					
HA-dp2	–	–	–	n.d.	n.d.
HA-dp3	–	–	–	n.d.	n.d.
psHA-dp2	4.5	–	–	n.d.	n.d.
psHA-dp3	4.3	112.9 ± 25.3	0.0	n.d.	n.d.
**Irreversible inhibitors**					
**7b**	–	2510 ± 140	0.0	n.d.	n.d.
**Z013**	–	60.0 ± 8.3	0.0	n.d.	n.d.

The obtained MC_50_ values in the nM range (Table 1) gave evidence that polymeric sulfated GAG derivatives are very potent in reducing TG2 crosslinking activity, comparable or even better than those of established irreversible inhibitors^42,43^ in our particular experimental setup (Supplementary Fig. S3). For example, for the commercially available TG inhibitor **Z013** (Zedira) an MC_50_ value of 60.0 ± 8.3 nM was calculated in our test system for gpTG2. The MC_50_ value of another recently published inhibitor (**7b**^40^) was calculated to be in the µM-range (2.51 ± 0.14 µM) in our setting. However, both inhibitors, **7b** and **Z013**, bind only in the presence of Ca^2+^ to TG2, which is only possible once the assay mix is added.

To exclude an interference of GAG with the assay procedure itself, e.g., by competing for poly-l-lysine coating on the assay plate and, therefore, causing a smaller availability of binding sites for TG2, a coating control experiment was performed exemplarily with SH3 and gpTG2: The concentrations (4.4 and 88 nM) were chosen according to the inhibition curves—a slight inhibition (20%) and a high inhibition (80%) would have been expected if there was an interference. However, activity of gpTG2 was not significantly impaired (Supplementary Fig. S4), suggesting that the inhibitory effect of sulfated GAG derivatives is not an artefact but a result of their interaction with TG2.

Furthermore, a “jump dilution” experiment was performed to get an idea on whether GAG are rather reversible or irreversible inhibitors according to Copeland^44^. Supplementary Fig. S5 shows that both HE and SH3 appear to be indeed reversibly bound to TG2, as the remaining activity after 100-fold dilution is at about 100% for HE and SH3. A control dilution sample (resulting in onefold concentration of enzyme and onefold MC_50_) showed roughly the expected activity (see also Table 1) with about 70% for HE and SH3.

### The inhibitory effect of sulfated GAG derivatives on TG2 activity requires a minimum sugar chain length

Due to the more pronounced inhibitory effect of gpTG2 in the employed readout and, therefore, easier handling in comparison to rhTG2, the guinea pig enzyme was used to further investigate the inhibitory mechanisms of sulfated GAG derivatives.

In order to determine a possible minimum sugar chain length (i.e. number of disaccharide units of GAG) for the GAG inhibitory capacity, non-sulfated and persulfated tetra- and hexasaccharides of HA (Supplementary Fig. S1, Table 3) were investigated. Neither the non-sulfated oligohyaluronans (HA-dp2 and -dp3) nor the persulfated psHA-dp2 affected gpTG2 activity (Fig. 1e–g). An appreciable dose-dependent effect on TG2 activity was only observed with the persulfated HA hexasaccharide psHA-dp3 (Fig. 1h), which indicates a minimum requirement of three disaccharide units for TG2 inhibition. The MC_50_ value of psHA-dp3 is with 112.9 ± 25.3 nM (Table 1) about one order of magnitude higher than for comparable polymeric sulfated GAG (i.e. SH3).

### The inhibitory effect of sulfated GAG derivatives and irreversible inhibitors is additive

The peptidic TG2 inhibitor **Z013**^42,45^ (Zedira GmbH) stabilizes the open conformation upon covalently binding to the catalytic site of TG2 (crystal structure of the TG2–Z013 complex: PDB ID 3S3P). Compound **7b** belongs to the chemotype of *N*^ε^-acryloyllysine piperazides^40^, which also inhibit TG2 in an irreversible manner. For structurally related inhibitors it has been shown by kinetic capillary electrophoresis that they also stabilize the open conformation^46,47^. Similar to the additional Ca^2+^ experiments (see below), an approach of subsequently adding GAG and the inhibitor **Z013** or **7b** (experimental setting III) was investigated.

**Figure 2:**
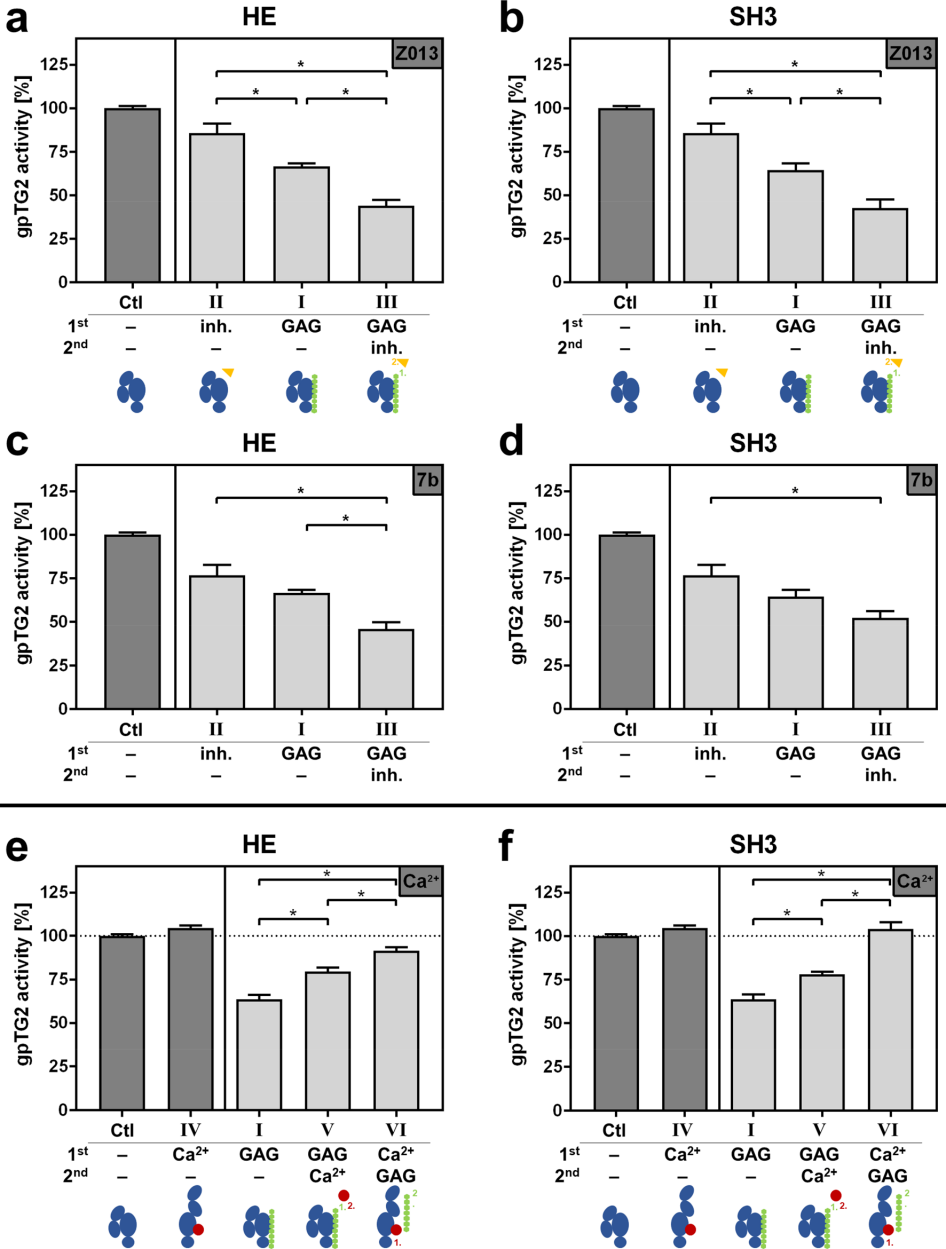
Influence of inhibiting or activating modifiers on the inhibitory effect of sulfated GAG derivatives. (**a**–**d**) Irreversible inhibitors: Before applying to the assay plate, gpTG2 was incubated according to experimental settings I–III with irreversible inhibitors **Z013** and **7b**, respectively (residual activity around 80%), and sulfated GAG derivatives (**a**,**c**) HE and (**b**,**d**) SH3, respectively (residual activity around 65%). Positive control “Ctl” (gpTG2 without any treatment) was set to 100%. (**e**,**f**) Ca^2+^ activation: gpTG2 was incubated according to experimental settings I (residual gpTG2 activity around 65%) and IV–VI with 5 mM CaCl_2_ and (**e**) HE or (**f**) SH3. The given Ca^2+^ concentration refers to that one in the reaction tube before the mixture was applied to the assay plate and assay buffer (with Ca^2+^ in excess) was added. Positive controls “Ctl” (gpTG2 without any treatment for “I”, “IV” and “V”; highlighted with a dotted line) and “IV” (gpTG2 activated with 5 mM CaCl_2_ concentration for “VI”) were set to 100%. Values in all panels are shown as mean ± SEM; n = 3. Significant differences (p < 0.05) between settings I–III (**a**–**d**) or I, V and VI (**e**,**f**) were calculated by one-way ANOVA and Bonferroni’s post-test and are indicated with *.

Figure 2a–d shows similar effects independently of using GAG or inhibitor. When gpTG2 was incubated with HE first and thereafter with both **Z013**/**7b** (experimental setting III), the inhibitory effect of both substances was additive, and even stronger in comparison to single treatment of either compound (HE/**Z013**: ~ 44%, HE/**7b**: ~ 46% remaining activity; Fig. 2a,c). Similar results were observed in the setup with SH3 and irreversible inhibitors (Fig. 2b,d). Experimental setting III led to a stronger inhibition (SH3/**Z013**: ~ 43%, SH3/**7b**: ~ 52% remaining activity) compared to experimental settings I and II.

### The inhibitory effect of polymeric sulfated GAG on TG2 is modulated by Ca^2+^

Ca^2+^ ions are needed to induce the conformational change of TG2 from the closed inactive to the open enzymatically active conformation^11,46^. In the experiments described before (using experimental setting I), TG2 and GAG were pre-incubated in the absence of Ca^2+^. Hence, activation did not occur before performing the TG2 activity measurement due to the included Ca^2+^-concentration in the assay buffer (> 85 mM, see Supporting Information “Calcium quantitation”). Therefore, the influence of prior addition of 2.5–20 mM Ca^2+^ to TG2 (experimental setting IV) was checked. Pre-activation (setting IV) did not change gpTG2 activity compared to Ctl (experimental setting I) and only to a low extent of rhTG2 (Supplementary Fig. S6).

To evaluate whether GAG interact preferentially with either closed or open TG2 conformation, a sequential approach was followed by adding first GAG and thereafter Ca^2+^ with each 5 min of incubation time (experimental setting V) or vice versa (experimental setting VI). Furthermore, experimental setting V served to check whether GAG are preventing an opening of the TG2 3D conformation by blocking Ca^2+^ binding sites of the enzyme. Figure 2e,f and Supplementary Fig. S7 show that the experimental settings V and VI performed with HE and SH3, each with rhTG2 and gpTG2, lead to a significant weakening of the inhibitory effect of both GAG. The inhibitory effect of HE on gpTG2 was almost completely abrogated (~ 92% activity) when Ca^2+^ was added first. In the same setting (VI) with SH3, the inhibitory effect was completely abolished. In setting V, the inhibitory efficiency was reduced, resulting in about 80% activity for both GAG. In fact, for setting V neither a lower (2.5 mM) nor a higher (20 mM) Ca^2+^ concentration altered this effect for SH3 on gpTG2 (Supplementary Fig. S8).

The obtained results highlight that the sequence order of the pre-incubation with sulfated GAG and Ca^2+^ is indeed crucial. They suggest a putative inhibition mechanism of GAG (see “Discussion” below).

### TG2 in closed conformation reveals manifold molecular recognition sites for GAG

**Figure 3:**
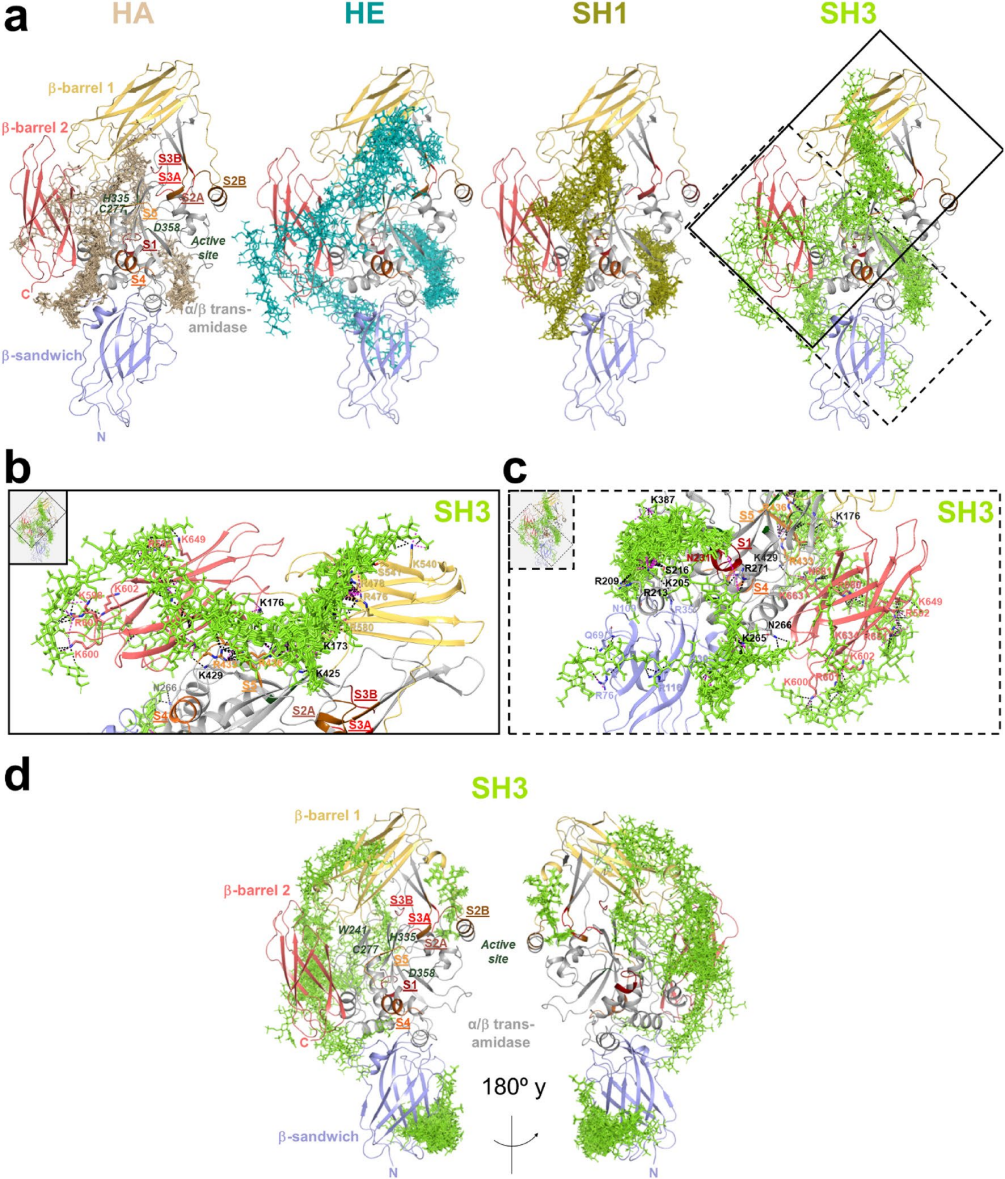
Molecular modeling of the interaction of the investigated GAG derivatives with TG2 in closed conformation. Docking results obtained using Glide for (**a**–**c**) GAG derivatives and rhTG2 as well as (**d**) SH3 and gpTG2. Protein domains are shown in cartoon: β-sandwich (purple), α/β-transamidase domain (gray), β-barrel 1 (yellow) and β-barrel 2 (salmon). Residues at the active site are highlighted in dark green. Ca^2+^ binding sites are highlighted in dark red (S1), brown (S2), red (S3) and orange (S4, S5). The different GAG clusters are shown in sticks: HA (pale), HE (teal), SH1 (smudge) and SH3 (green). The grid boxes (**b**) and (**c**) highlight the close-up view of the predicted interaction of SH3 with rhTG2 according to solid and dashed boxes in (**a**). Figure generated in *Maestro* (v12.3)^48^.

Molecular docking calculations were performed to predict and investigate putative GAG recognition sites at rhTG2 and gpTG2 along the entire protein surfaces. Both TG2 homologues share 83% and 91% sequence identity and similarity, respectively (Supplementary Fig. S11). For the first part of the in silico studies, both enzymes were considered in their closed conformation (Fig. 3), according to experimental setting I (Fig. 5). In order to cover the full protein structure in closed conformation, docking studies were performed in two main steps involving the β-sandwich, α/β-transamidase and β-barrel 2 domains and, on the other hand, the α/β-transamidase and β-barrels 1 and 2 domains. Hexasaccharidic GAG (as representatives of polymeric GAG) were predicted to bind along the four TG2 domains of both orthologous enzymes in the closed conformation (Fig. 3, Table 2).

**Table 2:** Predicted recognition sites for GAG hexasaccharides on rhTG2 closed conformation. ^a^Different recognition sites are shown in different columns or in the same column separated by dashed lines. ^b^Underlined residues correspond to low populated GAG recognition sites. ^c^It includes catalytic core (**cat**) (triad C277, H335, D358) and Ca^2+^ binding sites [**S1** (_226_GMVNCNDD_233_), **S2A** (_396_EVNADV_401_), **S2B** (_447_EGSSEERE_454_), **S3A** (_306_DQNSNLL_312_), **S3B** (_328_SEM_330_), **S4** (_151_DSEEERQE_158_) and **S5** (_433_RDERED_438_)]. ^d^Residues between contiguous domains are shown in italic.

β-sandwich^a,b^ (1–139)		α/β-transamidase^a,b,c^ (147–460)				β-barrel 1^a,b^ (472–583)		β-barrel 2^a,b,d^ (591–687)	
***HA***									
E15	R35	Q157 (**S4**)	P144	K173 (**cat**)	N229 (**S1**)	L555	R512	Q599	*E588*
R19	A108	E158	A145	K425	N231 (**S1**)	R580	L520	M659	*I589*
A24	N109	Q163	K202	D434 (**S5**)	G239			K600	R680
D25		Q164	R213	R436 (**S5**)	R271			L661	
		K429	S216		D326			K663	
		D434 (**S5**)	R222		K327			L688	
		––––	N231 (**S1**)		E329 (**S3B**)			––––	
		N243	T343		P361			Q633	
		Y245	P345		T368			K634	
		G246	K364					T635	
		G248	Y369					E637	
		R263	Y388					D653	
								K674	
***HE***									
––––	R35	––––	K202	K173 (**cat**)		R476		K600	R592
K30	N109	D198	R209	K176 (**cat**)		R478		R601	R651
T63	––––	R262	R213	K425		T496		––––	R680
P65		K265	S216	R433 (**S5**)		R580		K602	N681
K74		N266	R222	R436 (**S5**)				K634	
R116			N231 (**S1**)	D438 (**S5**)					
			R344						
			K364						
			K387						
			Y388						
***SH1***									
R19	R35	Q157 (**S4**)	K202	K173 (**cat**)	N231 (**S1**)	R476		Q599	R680
E29	E70	T162	R209	K176 (**cat**)	D232 (**S1**)	R478		M659	N681
K30	N109	Q163	R213	N177	D233 (**S1**)	G480		L661	
––––		K429	S216	K425	W241	N484		K663	
L26		R433 (**S5**)	R222	R433 (**S5**)	D242	R580		––––	
R28		––––	K364	D434 (**S5**)	R271	––––		Q633	
R116		R262	E366	R436 (**S5**)	D326	R512			
		R263	K387		S328 (**S3B**)	L520			
		K265	Y388		T368				
		N266							
***SH3***									
K30	R35	K429	K205	K173 (**cat**)		R476		K598	R592
H134	N109	––––	R209	K176 (**cat**)		R478		K600	K649
––––	––––	Q234	R213	N177		K540		R601	R651
	Q69	K265	S216	K425		S541		––––	K663
	K74	N266	R222	R433 (**S5**)		R580		K602	R680
	R76	R271	N231 (**S1**)	E435 (**S5**)				K634	N681
	R116		K387	R436 (**S5**)					
			Y388						

In the case of rhTG2, the investigated GAG (in major extent, the medium- and high-sulfated HE and SH3) were predicted to stabilize the closed conformation by acting as a “molecular staple” between the β-barrel 1, α/β-transamidase and β-barrel 2 domains (Fig. 3a). In β-barrel 1, all investigated GAG participated in interactions with R580 and, in addition, sulfated GAG derivatives recognized R476 and R478, which are constituents of the GTP binding site^49,50^. The common GAG recognition region along the α/β-transamidase domain involved residues not previously described as HE binding site: K173, K176, K425 and the reported S5 Ca^2+^ binding site^10^ through interactions with R433 and R436. Similarly, R680 from β-barrel 2 served as anchor recognition residue for all investigated GAG when bridging the α/β-transamidase domain. In the case of HE and SH3, the predictions revealed further interactions with R592, R680 and N681 (Fig. 3b,c, Table 2). The α/β-transamidase domain also served as anchoring of the GAG recognition site with the β-sandwich, and, to a lesser extent, the β-barrel 2 domain. Thus, docking predicted interactions of HA and SH1 with R19, Q157 (S4 Ca^2+^ binding site), Q163, K429 and D434 (for HA, S5 Ca^2+^ binding site), R433 (for SH1, S5 Ca^2+^ binding site)^10^, Q599 and K663. For SH3 and HE, the common interacting residues along the three domains were K30, K265, N266, K602 and K634 (Fig. 3c), which have been previously reported as recognition site of HS/HE^23,25^ (Supplementary Table S2). Also, two common recognition patterns of sulfated GAG bridging the β-sandwich and the α/β-transamidase domains were observed. The common recognition site involved residues R35, N109, K202, R213, S216, R222, N231 (S1 Ca^2+^ binding site), K364 and K387, among which K202, R213, S216 and R222 have been previously described as another HS/HE binding site (Supplementary Table S2)^25,26^. On the other hand, sulfated GAG participated in interactions with R262, K265, N266 and either with R28 (for SH1) or K30 (for SH3 and HE) (Fig. 3c), which resembles the previously reported HE binding site^23^.

The results obtained from molecular docking of SH3 on gpTG2 in the modeled closed conformation (see “Methods” for details) similarly suggested that the sulfated GAG derivatives could possibly stabilize the closed conformation through interactions between the α/β-transamidase domain and the β-barrels 1 and 2 and, to a lesser extent, the β-sandwich domain (Fig. 3d, Supplementary Table S3). A main gpTG2 recognition path by SH3 involved interactions with residues R30, R240, N244, R262, R263, K265, K553, R567, Q636, N670 and K677. Similarly, a second SH3 recognition path less populated was predicted distributed along residues R263, R271, K273, N318, N326, K327, K408, R567, T626, K634 and S638. As for rhTG2, SH3 participated in interactions with the β-barrel 1 residues R481, Q484 and T487 (the rhTG2/gpTG2 residue correspondences are as follows: R478/R481, Q481/Q484 and N484/T487). However, these binding poses did not interconnect with the α/β-transamidase domain as predicted for the human analogue. Furthermore, in contrast to rhTG2, only one SH3 recognition site along the β-sandwich residues Y50, R76, S78, S80, S81 and S129 was observed (Supplementary Table S3).

## Discussion

Previous studies have shown controversial results on how GAG recognition affects TG2 activity^26,37,38^. The present work sought to address exactly this topic—evaluating the inhibitory potential of GAG towards the crosslinking activity of TG2 by combining different experimental set-ups with in silico molecular docking techniques.

The data obtained by an activity-based assay (incorporation of Biotin-TVQQEL-OH onto poly-l-lysine coated 96-well plates) clearly showed that polymeric GAG reduce the crosslinking activity of TG2 in a concentration-dependent manner with MC_50_ values in the double to triple-digit nM range, when both were pre-incubated in the absence of Ca^2+^. In fact, the inhibitory effect was restricted to sulfated GAG derivatives (Fig. 1a–d). In this context, the interaction of positively charged l-lysine on the assay plate with the negatively charged sulfate groups of these GAG was ruled out as reason for the reduced enzyme activity (Supplementary Fig. S4). The D_S_ did not seem to influence the inhibitory effect, considering that the MC_50_ values for SH1 and SH3 were in close range for gpTG2 and rhTG2. The inhibitory effect of sulfated GAG required a minimum number of three disaccharide units (Fig. 1e–h). However, polymeric GAG are more potent for inhibiting TG2, as the psHA hexasaccharide was a much weaker inhibitor than its polymeric analogue SH3 (MC_50_ psHA-dp3 ~ 113 nM versus SH3-polymer ~ 17 nM towards gpTG2). A further experimental setting was tested to investigate the influence of known TG2 inhibitors (**Z013** and **7b**) on the inhibitory behavior of sulfated GAG toward TG2 (Fig. 2a–d). As shown, treatment of TG2 with SH3 or HE followed by **Z013** or **7b** enhanced the inhibitory effect compared to single treatment of TG2. The distinct additivity can be explained by considering the Ca^2+^-dependent binding of **Z013** and **7b**. Sulfated GAG bind to TG2 when they are added first (without Ca^2+^). Upon addition of Ca^2+^, activated TG2 (open conformation) is rapidly targeted by the irreversible inhibitors which ultimately leads to a stronger overall inhibitory effect on the transamidase activity. Therefore, TG2 transamidase activity was tested in additional settings with respect to Ca^2+^ activation and its influence on the inhibitory effect of GAG. The prior addition of Ca^2+^ (setting VI) significantly decreased the inhibitory effect of SH3 and HE on TG2 activity (Fig. 2e,f and Supplementary Fig. S7). In case of gpTG2, a pre-activation with Ca^2+^ before adding the sulfated GAG even prevented almost completely the inhibitory effect (Fig. 2e,f). Therefore, it can be proposed that the sulfated GAG reversibly inhibit TG2 and exert their inhibitory effect exclusively in the absence of Ca^2+^, and the total transamidase activity is restored in a time-dependent manner after addition of Ca^2+^ in excess. Considering that the applied activity assay uses an endpoint readout (30 min), the distinct residual enzymatic activity of rhTG2 at high concentrations of GAG is comprehensible. A “jump dilution” experiment^44^ with SH3 and rhTG2 was performed that further proved the reversible inhibition of TG2 by sulfated GAG (Supplementary Fig. S5). In accordance with these findings, Scarpellini et al*.*^38^ previously did not observe any influence of HE on TG2 activity. As reported by their experimental protocols, activity of TG2-containing cell lysates was detected without pre-activation/-incubation steps, but with a simultaneous addition of Ca^2+^ and HE.

TG2 can adopt different conformations—in fact a closed conformation upon binding of guanine nucleotides^50^, which is also the dominant conformation in the absence of any regulators^46^, and an open conformation in the presence of Ca^2+^^46^ and in complex with irreversible inhibitors^11^. Considering these available information, the sulfated GAG might bind to TG2 in closed conformation. To support the experimentally observed GAG-induced inhibitory effects and to provide atom-detailed insights into the GAG-TG2 interaction sites and residues involved, in silico docking calculations were performed using 3D molecular models based on the closed conformation of TG2. In silico based predictions were performed with GAG hexasaccharides instead of the experimentally used polymers. It was previously shown by the Pisabarro group that, when analyzing clusters of binding poses, oligomeric GAG can be taken as representative for investigating the interaction and binding modes of polymers^51^. Thus, the efficiency of the longer polymers to simultaneously impair multiple binding sites without presenting some sterically clashes would be higher than for the shorter oligomeric GAG. The closed rhTG2 and gpTG2 theoretical models predicted GAG binding regions distributed along the four protein domains in both enzymes. Furthermore, they highlighted that binding of sulfated GAG to TG2 is mainly based on electrostatic interactions between acidic functional groups of the polysaccharide and basic residues of the protein supported by the formation of binding clusters at the protein surface. Moreover, several polar uncharged TG2 residues were predicted to interact with GAG, which might imply specificity in GAG recognition^52^. In particular, GAG appeared to act as a “molecular staple” towards the closed conformation of TG2 by bridging the α/β-transamidase with the β-barrels domains resulting in a stabilization of the closed conformation. Moreover, a shared characteristic among the GAG is a potential interaction with R580 (β-barrel 1), which has been reported as crucial residue for binding of guanine nucleotides^49,53^, in a mode that bridges this residue with the S5 Ca^2+^ binding site, which has not been previously described as an HE binding site. GTP and analogues therefore induce and stabilize the closed conformation. However, only sulfated GAG can be recognized by TG2 through interactions with R476 and R478, which also contribute to GTP binding^50^. Thus, the observed effects of the GAG on the transamidase activity could additionally originate from the interaction with R580 in combination with R476 and R478.

In addition to the GAG binding poses observed, there were also binding poses predicted in which GAG binding occurs on a single TG2 domain. In this context, for GAG hexasaccharides, binding poses along the β-barrel 2 partially resembled the previously reported HS/HE binding site by Lortat-Jacob et al. in the same TG2 conformation^23^. At the α/β-transamidase domain, binding poses of sulfated GAG interacting with R262, R263, K265 together with R28 at the β-sandwich domain were predicted, which resembles a previously reported HE binding site^23,25^. In addition, the predicted GAG interactions with the basic residues K202, K205, R209, R213 and R222, also at the α/β-transamidase domain, are fully in agreement with HS/HE binding sites previously described by Teesalu et al. and Wang et al. in the closed conformation^25,26^. These residues constituted a path for GAG recognition that bridges the β-sandwich and the α/β-transamidase domains.

All investigated GAG derivatives were also predicted to interact with the β-sandwich domain to a different extent. In addition to their potential “molecular staple” function, the binding to the β-sandwich domain could explain their inhibitory action on the transamidase activity. Kim et al.^54^ recently demonstrated that the small molecule GK921 (a pyrido[2,3-*b*]pyrazine derivative) exhibits an MC_50_ value of 8.93 µM towards gpTG2 (pre-incubation in the absence of Ca^2+^) and binds to the β-sandwich domain of TG2. Inhibition of TG2 was shown to be a result of non-covalent multimerization as a consequence of conformational changes triggered by binding of GK921.

Furthermore, sulfated GAG derivatives are suitable to recognize different Ca^2+^ binding sites of TG2 (Table 2). All investigated GAG were able to recognize the rhTG2 S5 Ca^2+^ binding site as well as S1. Furthermore, S4 and S3B interacted with HA and its low-sulfated derivative SH1. The S1 Ca^2+^ binding site, although representing a strong recognition site, is not determinant for TG2 activity. However, for S5 a cooperative role with S3 has been proposed^10^. Therefore, it is also plausible that GAG unfold their inhibitory effect by hindering Ca^2+^ binding to TG2 closed conformation.

Molecular docking was also performed considering the open conformation of TG2 in an unbiased manner (Supplementary Results and Discussion, Supplementary Figs. S9–S11, Supplementary Tables S4, S5). Overall, GAG might recognize the catalytic core as well as different Ca^2+^ binding sites, although they seem to prefer the closed conformation over the open form of the enzyme. Interestingly, all investigated GAG were able to recognize K173 of the catalytic core when the enzyme was considered in closed conformation. Additionally, the presented theoretical models predict that only sulfated GAG (HE, SH1, and SH3) could recognize the exposed residue K176 of the TG2 catalytic core (Table 2). However, in open conformation only sulfated GAG, in contrast to the non-sulfated HA, still exhibited interactions with both residues. Therefore, interactions of GAG might also be possible with TG2 adopting an open conformation. Further structural studies would be required in order to get deeper insights on GAG recognition by TG2 in the closed and open conformations. However, according to the TG2 activity assay, if binding of the GAG occurs in the presence of Ca^2+^, this does not interfere with the transamidase activity. In this context, recent reports showed the preferred binding of HS/HE to the closed conformation of TG2 via different experimental techniques assessing the binding of TG2 in defined conformations to immobilized HE^23,26,55^.

Overall, the observations obtained with the molecular models complement the results obtained from the activity-based assay, which allows to conclude that the sulfated GAG bind to TG2 in closed conformation, stabilize this structural form and induce by this action the inhibitory effect on the transamidase activity.

## Conclusions

**Figure 4:**
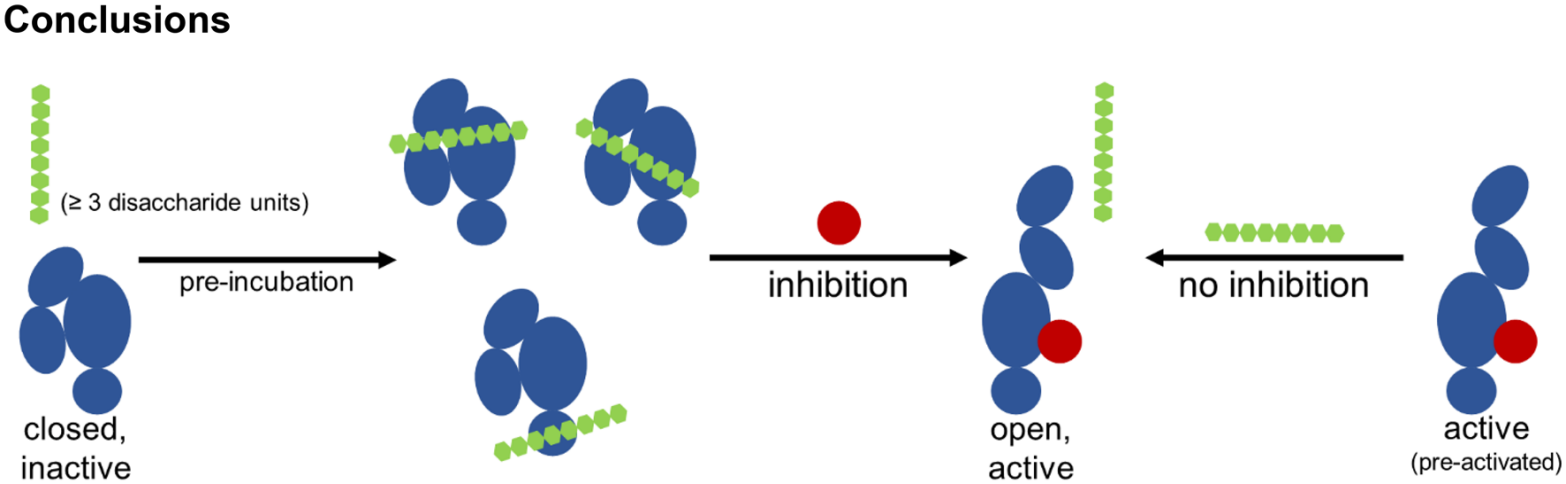
Schematic overview of the results and proposed mechanisms. When inactive TG2 (closed form) is pre-incubated with sulfated GAG derivatives in the absence of Ca^2+^, inhibition of transamidase activity is possible. Thereby, sulfated GAG presumably act as a “molecular staple” and stabilize the closed conformation of TG2. Furthermore, they might occupy the N-terminus (and induce conformational changes) as well as Ca^2+^ binding sites. When sulfated GAG are incubated with Ca^2+^-activated TG2 (open form), no inhibition of transamidase activity is observed.

This study shows for the first time that synthetically sulfated GAG derivatives reveal inhibitory effects on TG2 crosslinking activity. Importantly, the observed inhibitory effect of the GAGs critically depends on their binding to TG2 in the absence of Ca^2+^ ions. The minimum length of sulfated GAG derivatives showing any effect on TG2 activity was determined to be three disaccharide units. All polymeric sulfated GAG derivatives reduced TG2 activity in a concentration-dependent manner with calculated MC_50_ values in the nM range, whereas non-sulfated HA did not affect enzyme activity. The proposed theoretical models predict GAG recognition sites along the four TG2 domains in closed and open conformations and mostly involve electrostatic interactions with TG2 basic residues and the formation of binding clusters at the protein’s surface. Sulfated GAG derivatives were predicted to recognize the reported Ca^2+^ binding sites located at the TG2 α/β-transamidase domain. Based on the presented investigations, several natural and synthetically sulfated GAG derivatives with a high inhibitory potential against TG2 were identified. Overall, a molecular mechanism for their inhibitory function was proposed based on their ability to compete for crucial Ca^2+^ binding sites and to stabilize a closed conformation of TG2 (Fig. 4). These findings might bear physiological implications as TG2 in its closed conformation is proposed to act as a prominent adhesion co-receptor for fibronectin in complex with integrins and syndecan-4^56,57^. Therefore, the interaction of GAG derivatives with TG2 could either support its function as extracellular adapter protein or prevent it^36,58^, even though this remains to be proven experimentally. Further physiological relevance of the GAG-TG2 interaction is given by the syndecan-4-dependent translocation into the extracellular space, which is initiated by the binding of TG2 to the HS epitopes of vesicle-associated intracellular syndecan-4^57,59^. Although the open conformation of TG2 (and therefore its transamidase activity) in the extracellular environment is probably not targeted by sulfated GAG derivatives, their interaction with TG2 in the closed conformation state could potentially already occur before the enzyme is released into the ECM since sulfated GAG derivatives have been shown to be internalized by various cell types^60–62^. In this light, the obtained results encourage further studies focusing on the applications of GAG derivatives towards the treatment of clinically relevant diseases in which TG2 is involved, such as cancer and fibrosis.

## Materials

Purified guinea pig TG2 (gpTG2) was purchased from Sigma Aldrich (Taufkirchen, Germany; T5398-1U; provided in the kit CS1070). Purified recombinant human TG2 (rhTG2; #T022) and the TG2 inhibitor **Z013** were obtained from Zedira (Darmstadt, Germany). The TG2 inhibitor **7b** was synthesized as described recently^40^ (same numbering as in^40^). Characteristics of the inhibitors are summarized in Table 3 and formulas are depicted in Supplementary Fig. S1.

**Table 3:** Characteristics of the investigated polymeric and oligomeric GAG derivatives. ^a^ MW, molecular weight (weight-average molecular weight determined by gel permeation chromatography combined with a laser light scattering detector); ^b^ D_S_, degree of sulfation (average number of sulfate groups per disaccharide repeating unit); *HA with low MW obtained after thermal degradation; dp (degree of polymerization).

	Abbreviation	MW^a^ (kDa)	D_S_^b^	Position of sulfation within disaccharide unit	References
**Polymeric GAG**					
Hyaluronan*	HA	146	–	None	^63^
Heparin	HE	19	2.2	Irregular	^36,64^
Low-sulfated HA	SH1	31	1.2	C6, C2′, C3′	^36,64,65^
Medium-sulfated HA	SH2	18	2.0	C4, C6, C2′, C3′	^65^
High-sulfated HA	SH3	57	3.2	C4, C6, C2′, C3′	^65^
Low-sulfated chondroitin-sulfate	CS1	20	1.0	C4 (70%), C6 (30%)	^64,66^
Medium-sulfated CS	CS2	23	1.8	C4, C6, C2′, C3′	^66^
High-sulfated CS	CS3	32	2.8	C4, C6, C2′, C3′	^66^
**Oligomeric GAG**					
HA-tetrasaccharide	HA-dp2	0.80	–	None	^67^
HA-hexasaccharide	HA-dp3	1.18	–	None	^68^
Persulfated HA-tetrasaccharide	psHA-dp2	1.76	4.5	C4, C6, C2′, C3′	^67^
Persulfated HA-hexasaccharide	psHA-dp3	2.57	4.3	C4, C6, C2′, C3′	^68^
**Irreversible TG2 inhibitors**					
**7b**	–	0.480	–	–	^40^
**Z013**	–	0.688	–	–	^45^

Native CS (CS1, isolated from porcine trachea) was obtained from Kraeber (Ellerbeck, Germany), HE (from porcine intestinal mucosa) from Sigma Aldrich (Taufkirchen, Germany), and high molecular weight (MW) HA (isolated from *Streptococcus*, MW = 1200 kDa) from Aqua Biochem (Dessau, Germany).

All chemicals, if not stated otherwise, were purchased from Sigma Aldrich (Taufkirchen, Germany).

## Methods

### Preparation and characterization of polymeric and oligomeric GAG derivatives

Polymeric GAG derivatives with low, medium and high degree of sulfation (D_S_) based on HA and CS were synthesized as described^69^. The values for D_S_ (average number of sulfate groups per disaccharide repeating unit) were determined by elemental analysis and nuclear magnetic resonance (NMR) and MW was determined by laser light scattering^66^. The chemical structures and characteristics of respective GAG derivatives are given in Supplementary Fig. S1 and Table 3.

Oligomeric non-sulfated and persulfated HA derivatives were synthesized according to^67,68^. Briefly, tetra- (HA-dp2) and hexahyaluronan (HA-dp3) derivatives were obtained after hyaluronidase treatment of high-MW HA. Resulting products were separated by size exclusion chromatography. Anomeric fixation (β-configuration) with an azide moiety and subsequent persulfation to the nona-sulfo-tetra- (psHA-dp2) and trideca-sulfo-hexahyaluronan (psHA-dp3) have been described before^67,68^. The final products were analyzed by ^1^H and ^13^C NMR spectroscopy and by electrospray ionization mass spectrometry to determine the D_S_. The chemical structures and characteristics of oligomeric HA derivatives are summarized in Supplementary Fig. S1d and Table 3.

### Determination of TG2 enzymatic activity

**Figure 5:**
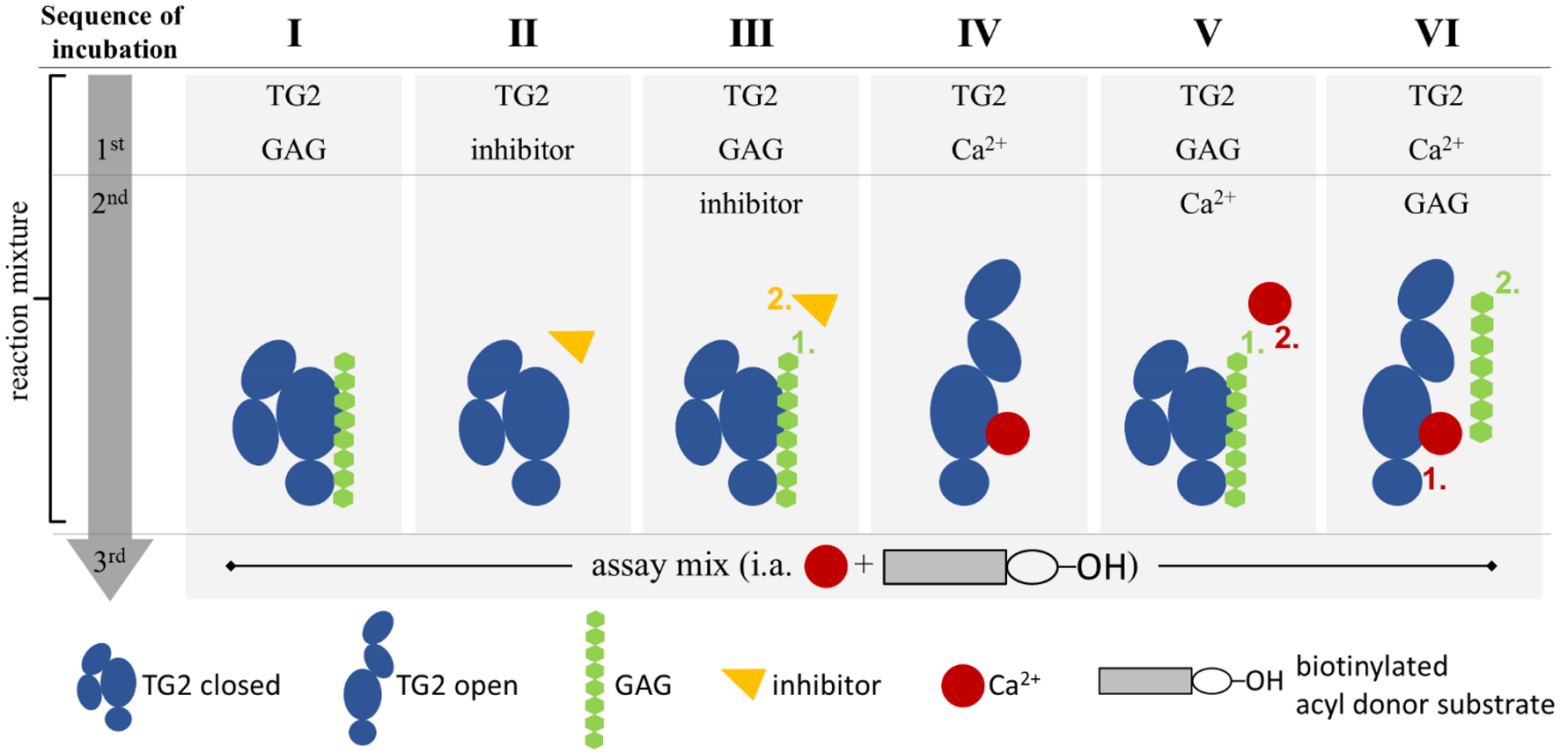
Experimental setup of TG2 enzyme activity assay. For crosslinking activity determination of TG2 orthologues a colorimetric activity assay kit (CS1070, Sigma Aldrich) was used. By varying the incubation steps (I–VI; 5 min each) before the reaction mixture was applied to the poly-l-lysine coated assay plate, cooperative effects with irreversible inhibitors (II–III) as well as the influence of sulfated GAG with and without Ca^2+^ (I, IV–VI) were investigated.

TG2 crosslinking activity was determined using a peroxidase-coupled colorimetric activity assay kit (CS1070, Sigma Aldrich) according to manufacturer’s instructions. It is based on the TG2-catalyzed incorporation of Biotin-TVQQEL-OH, which acts as acyl donor substrate, onto a poly-l-lysine coated 96-well plate. Prior to the assay, gpTG2 was dissolved to 2 U/mL in 10 mM DTT with 1 mM EDTA, stored at 4 °C and used within two weeks. rhTG2 was dissolved according to manufacturer’s instructions to ~ 1.19 mg/mL (> 1500 U/mg) in H_2_O and stored until use at − 80 °C. If not stated otherwise, both enzymes were used in the tests without any pre-activation. There is an abundant amount of Ca^2+^ present in the actual assay buffer and, thus, the assay mix, causing the “opening” of TG2 (= activation), and, therefore, enabling the enzyme to crosslink. The different experimental settings are depicted in Fig. 5 and described below in detail.

#### Influence of polymeric and oligomeric GAG derivatives (experimental setting I)

To study the influence of GAG on TG2 activity, the enzyme solution was incubated with GAG (as activity “modifier”; diluted with dH_2_O) for 5 min at 25 °C before applying 50 µL of the reaction mixture (containing 0.1 mU gpTG2; 39.7 ng rhTG2 and the desired amount of GAG) to the assay plate. After adding 50 µL of assay mixture (containing the substrate and Ca^2+^) to each well, samples were incubated for 30 min at 25 °C. Afterwards, streptavidin-peroxidase solution (1 µg/mL) was freshly prepared, added and incubated for 20 min. The reaction with 3,3′,5,5′-tetramethylbenzidine substrate (included in the kit, no further dilution required) was stopped after 1–2 min followed by detection of absorbance at 450 nm with a plate reader (Benchmark Plus, BioRad). Negative and positive controls (H_2_O and TG2 without any GAG, respectively) were always run in parallel. Each sample was measured in duplicate. For calculation, the mean signal of the negative control was used as a blank and subtracted from further data of the same experiment. Afterwards, the signal of positive control (pure TG2) was set to 100% and the signal of TG2 in presence of GAG was related accordingly. The final concentration of GAG in the whole reaction (considering the total volume of the reaction mixture with assay mix) was used for the determination of the MC_50_ values^41^ (see “Results” section for definition). Non-linear fit calculation was performed using models of dose–response curves as implemented in the *GraphPad Prism 8.02* software. The calculated MC_50_ values are given in the plots. In case of complete inhibition to 0% activity, “[inhibitor] versus normalized response—variable slope” was used, expressed as

1 $${\text{Y}}=\frac{{{100}}}{{{1 }+\left( {\frac{{{\text{MC}}_{{{50}}} }}{{\text{X}}}} \right)^{{{\text{HillSlope}}}} }}$$

whose MC_50_ corresponds to 50% activity. When complete inhibition to 0% activity was not possible, “[inhibitor] versus response—variable slope” was used instead, expressed as

2 $${\text{Y}} = {\text{Bottom}}+ \frac{{\text{Top-Bottom}}}{{{1 }+\left( {\frac{{{\text{MC}}_{{{50}}} }}{{\text{X}}}} \right)^{{{\text{HillSlope}}}} }}$$

with “activity_bottom_” and “activity_top_” representing the lower and upper plateaus of the sigmoid dose–response curve, respectively.

#### Coating control experiment

The assay plate was pre-incubated with GAG for 5 min at 25 °C and then washed briefly with H_2_O before TG2/assay mix without any further inhibitory compounds was added.

#### “Jump dilution” experiment with TG2 and GAG

To evaluate the putative GAG inhibition mode, the following “jump dilution” experiment^44^ was performed: Samples were prepared by incubating rhTG2 enzyme (20-fold: 7.9 µg/mL; 200-fold: 79.4 µg/mL) with 20-fold MC_50_ of SH3 and HE (see Supplementary Table S1 for details) for 5 min at 25 °C. Afterwards, they were diluted in H_2_O to 2-fold or 0.2-fold MC_50_ before adding them to the assay plate. The samples were further diluted with assay mix to adjust the amount of enzyme per well to that used in the tests before (rhTG2: 39.7 ng) as well as variations of the MC_50_ concentration of SH3 (either 1-fold MC_50_ or 0.1-fold MC_50_). Depending on how the activity of TG2 was shifted, the inhibition mode (reversible or irreversible) could be stated. According to Copeland^44^, the inhibitory mechanism can be determined by incubating the enzyme at 100-fold concentration with inhibitor at a concentration equal to 10-fold of its MC_50_ value before diluting it to the 1-fold concentration of enzyme resulting in a 0.1-fold concentration of MC_50_. If the activity measured afterwards equals about 91% activity of the positive control (assuming a possible 100% inhibition), a rapidly reversible mechanism could be concluded.

#### Influence of irreversible inhibitors (experimental setting II)

Inhibitors with known molecular mechanisms, **Z013**^45^ and **7b**^40^, were used in the same experimental setting as described above for GAG (experimental setting I). In order to compare and evaluate the effects achieved with GAG, TG2 was not pre-activated with Ca^2+^ and came only in contact with the divalent cation, when assay mix was added.

#### Competitive approach of sulfated GAG and irreversible inhibitors (experimental setting III)

To further characterize the inhibitory mechanism of GAG by investigating their possible competition with irreversible inhibitors for binding sites, a different incubation approach was used. TG2 was pre-incubated with sulfated GAG in concentrations that result in TG2 activities of 65% (sulfated GAG). This was followed by the addition of inhibitor (**7b**, **Z013**), whose concentration results in 80% residual activity in setting II. Each incubation step was performed for 5 min at 25 °C.

#### Influence of Ca^2+^ on sulfated GAG-induced inhibition of TG2 activity (experimental setting IV–VI)

Ca^2+^ ions are mandatory to activate TG2. To answer the question whether sulfated GAG interact preferentially with the open or closed TG2 conformation, the enzyme was incubated with 5 mM CaCl_2_ prior or after GAG addition (experimental setting VI or V, respectively). The experiments were performed with a supplementary amount of GAG that results in a TG2 activity of about 65% to better visualize putative additive inhibitory effects. For the sequential settings, each incubation step was performed for 5 min at 25 °C.

### Statistics

Three experiments were performed, each with separately prepared duplicates (resulting in 6 values). Statistical analyses were conducted with the *GraphPad Prism 8.02* software. Outliers were identified via ROUT method (Q = 5%) and removed. If both values of one concentration in one experiment were detected as outliers, the according concentration was repeated in another experiment to complete the data set. Statistical significance (p < 0.05) was analyzed by either *t* test or one-way ANOVA with Bonferroni’s post-test as indicated in the diagrams.

### Molecular modeling

#### Protein modeling

To model the three-dimensional (3D) structure of gpTG2 in closed conformation, the crystal structure of rhTG2 (https://www.rcsb.org/; PDB ID 3LY6^70^, 3.1 Å) was used as template (83% sequence identity, 91% sequence similarity). *Modeller* was used as implemented in *Discovery Studio*^71^. Modeling of the 3D structures in open conformation has been described in our previous work^40^.

#### Molecular docking

Prior to the docking studies, the proteins and ligands were prepared as follows:

##### Proteins

rhTG2 and the modelled gpTG2 structures in closed and open conformation were prepared in the *Protein Preparation Wizard*^72^ from Schrödinger. The structures of rhTG2 and gpTG2 in open conformation, and keeping the H335 in its protonated state as previously reported^40^, were further energy-minimized using the *OPLS3e* force field^73^ and a converge criteria of RMSD 0.3 Å for heavy atoms.

##### Ligands

Hexasaccharides of HA, SH1 (sulfated at C6), SH3 (sulfated at C3′, C4 and C6) and HE were prepared with *LigPrep*^74^ in the *Maestro suite*^48^. *Epik*^75,76^ was used to generate an ionization state at pH 7.0 ± 2.0. The *OPLS3e* force field was used for their energetic optimization^73^.

Blind docking studies were then carried out for each protein in closed and open conformation using *Glide*^77,78^ in standard precision. In the case of TG2 in closed conformation, two docking experiments involving either the α/β-transamidase and the two β-barrel domains or the α/β-transamidase, β-barrel 2 and the β-sandwich domains were carried out (see below for details on the grid boxes used). In the case of TG2 in open conformation, docking was performed on each protein domain. In addition, a partial overlap of contiguous domains to the main protein domain considered for docking runs was ensured (see below for details on the grid boxes used). Ligands were considered flexible. In proteins, dihedrals of the side chains of residues C, S, T and Y were allowed to rotate. For rhTG2 in closed conformation, the grid boxes were set up around Q96 and Y125 (β-sandwich domain), R377 and with or without including K205 (α/β-transamidase domain) in order to fully cover all regions of the α/β-transamidase domain with the β-sandwich, R592 and L688 (β-barrel 2 domain) as well as around K205 (α/β-transamidase domain), K468 and N531 (β-barrel 1 domain) and R592 (β-barrel 2 domain). For gpTG2 closed conformation, the grid boxes were set up similarly around Q96, Y125 (β-sandwich domain) and, as explained above for rhTG2, with or without including K205, R377 (α/β-transamidase domain), R595 and A690 (β-barrel 1 and 2 domains, respectively) and around K205 (α/β-transamidase domain), K467, N534 (β-barrel 1 domain) and R596 (β-barrel 2 domain). In addition, another grid box was set up around R271, R432 (α/β-transamidase domain), R515 (β-barrel 1 domain), R595 and R604 (β-barrel 2 domain). For rhTG2 in open conformation, the grid boxes were set up for the β-barrel 2 domain around D409, D553, K600, K602, E632 and E637, for the β-barrel 1 domain around N243, L462, E539 and K674, for the α/β-transamidase domain around E154, K173, L204, I323 and Q348, and for the β-sandwich domain around C98, Y125, I255 and P345 with an inner box of 40 Å × 40 Å × 40 Å and an outer box of 76 Å × 76 Å × 76 Å. Similarly, the grid boxes for the gpTG2 open conformation were set up for the β-barrel 2 domain around D409, D556, N603, K605, D635 and E640, for the β-barrel 1 domain around N243, L461, E542 and K677, for the α/β-transamidase domain around Q154, K173, L204, I323 and E348, and for the β-sandwich domain around S98, Y125, I255 and P345. The *OPLS3e* force field was used^73^. Docking results were ranked according to the obtained docking score.

## Supplementary Information

Supplementary Information.

## Data Availability

Correspondence and requests concerning original scientific data sets should be addressed to the corresponding authors M.T.P and S.V.
