# Author Correction: Sulfated glycosaminoglycans inhibit transglutaminase 2 by stabilizing its closed conformation

**DOI:** 10.1038/s41598-022-21032-7

**Published:** 2022-09-28

**Authors:** Claudia Damaris Müller, Gloria Ruiz-Gómez, Sophie Cazzonelli, Stephanie Möller, Robert Wodtke, Reik Löser, Joanna Freyse, Jan-Niklas Dürig, Jörg Rademann, Ute Hempel, M. Teresa Pisabarro, Sarah Vogel

**Affiliations:** 1 Institute of Physiological Chemistry, Medical Faculty Carl Gustav Carus, Technische Universität Dresden, Fetscherstraße 74, 01307 Dresden, Germany; 2 Structural Bioinformatics, BIOTEC, Technische Universität Dresden, Tatzberg 47-51, 01307 Dresden, Germany; 3 Biomaterials Department, INNOVENT E.V., Prüssingstraße 27 B, 07745 Jena, Germany; 4 Helmholtz-Zentrum Dresden-Rossendorf, Institute of Radiopharmaceutical Cancer Research, Bautzner Landstrasse 400, 01328 Dresden, Germany; 5 Institute of Pharmacy, Freie Universität Berlin, Königin-Luise-Straße 2/4, 14195 Berlin, Germany

Correction to: *Scientific Reports* https://doi.org/10.1038/s41598-022-17113-2, published online 03 August 2022

The original version of this Article contained errors in Table 2, where the corresponding footnote ‘b’ was omitted in the heading row for ‘β-sandwich (1–139)’, β- ‘barrel 1 (472–583)’ and ‘β-barrel 2 (591–687)’. The correct and incorrect values appear below.

Incorrect:

**Table:** 

**β-sandwich**^**a**^** (1–139)**	**β-barrel 1**^**a**^** (472–583)**	**β-barrel 2**^**a,d**^** (591–687)**

Correct:

**Table:** 

**β-sandwich**^**a,b**^** (1–139)**	**β-barrel 1**^**a,b**^** (472–583)**	**β-barrel 2**^**a,b,d**^** (591–687)**

The original Article has been corrected.

