# Supplementary material for: Sulfated glycosaminoglycans inhibit transglutaminase 2 by stabilizing its closed conformation
Source: Sci Rep. 2022 Aug 3;12:13326. doi: 10.1038/s41598-022-17113-2 (PMC9349199; doi:10.1038/s41598-022-17113-2)
Supplement: Supplementary file 1 — Supplementary Information. [file 41598_2022_17113_MOESM1_ESM.pdf]

Sulfated glycosaminoglycans inhibit transglutaminase 2 by stabilizing its closed conformation.

Claudia Damaris Müller[1#], Gloria Ruiz-Gómez[2#], Sophie Cazzonelli[1], Stephanie Möller[3], Robert Wodtke[4], Reik Löser[4], Joanna Freyse[5], Jan-Niklas Dürig[5], Jörg Rademann[5], Ute Hempel[1], M. Teresa Pisabarro[2*], Sarah Vogel[1*]

[1] Institute of Physiological Chemistry, Medical Faculty Carl Gustav Carus, Technische Universität Dresden, Fetscherstraße 74, 01307 Dresden, Germany.
[2] Structural Bioinformatics, BIOTEC, Technische Universität Dresden, Tatzberg 47-51, 01307 Dresden, Germany.
[3] Biomaterials Department, INNOVENT e.V. Prüssingstraße 27 B, 07745 Jena, Germany.
[4] Helmholtz-Zentrum Dresden-Rossendorf, Institute of Radiopharmaceutical Cancer Research, Bautzner Landstrasse 400, 01328 Dresden, Germany.
[5] Institute of Pharmacy, Freie Universität Berlin, Königin-Luise-Straße 2/4, 14195 Berlin, Germany.

*Corresponding authors: maria_teresa.pisabarro@tu-dresden.de, sarah.vogel@tu-dresden.de
# Both authors contributed equally to this work.

Supplementary Information

Supplementary Materials and Methods

Polymeric and oligomeric GAG derivatives and irreversible inhibitors.

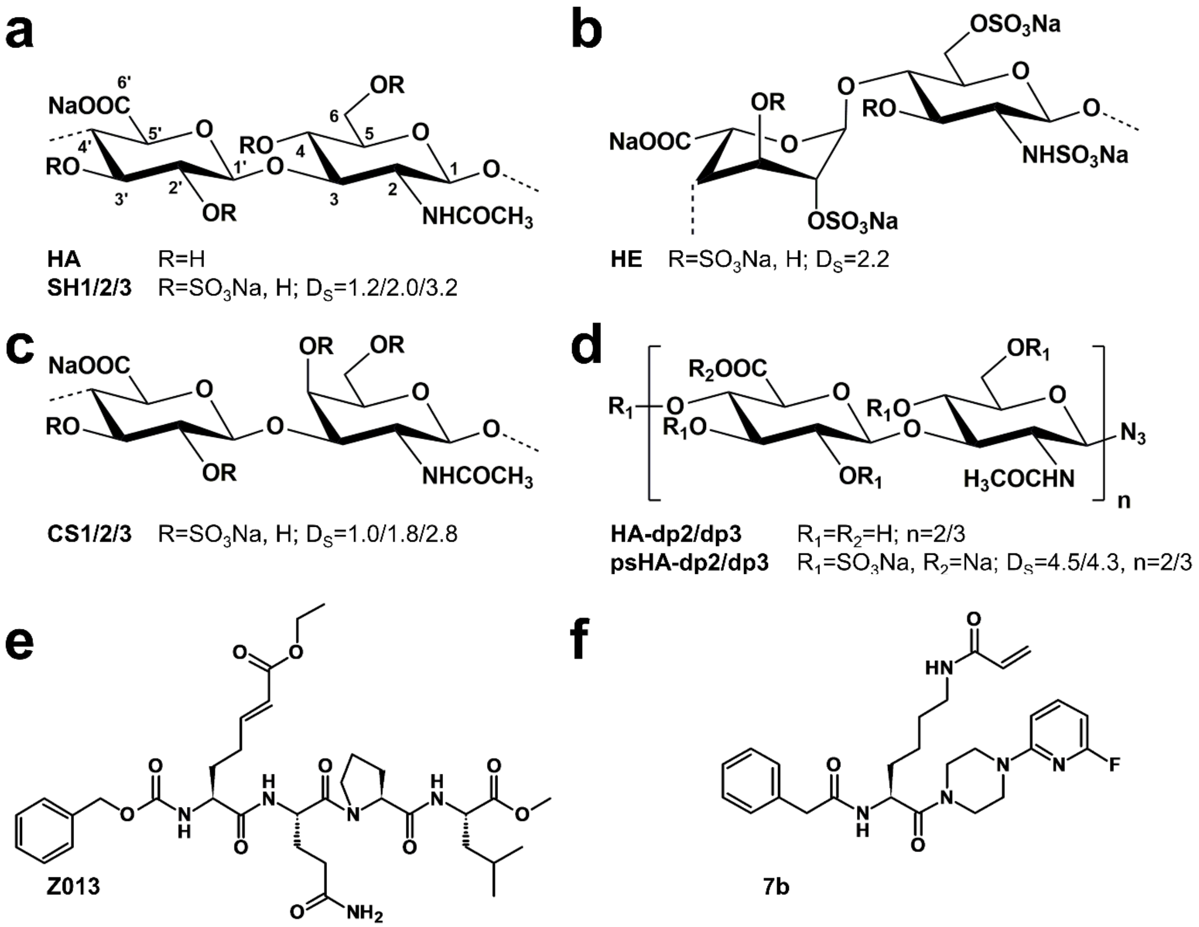

Figure S1. Chemical structures of polymeric and oligomeric GAG derivatives and irreversible inhibitors.
Formulas of **a**) HA and its SH1/2/3 derivatives, **b**) HE, **c**) CS1/2/3, **d**) oligomeric HA-dp2/dp3 and psHA-dp2/dp3, and TG2 inhibitors **e**) Z013 and **f**) 7b.

Calcium quantitation.

Ca^{2+} concentration of assay buffer was determined with the calcium kit (Greiner Diagnostics, Bahlingen, Germany) as previously described[1]. Briefly, 2 µl assay buffer were quantified photometrically with 200 µl cresolphthalein complexone at 570 nm. Ca^{2+} content was calculated from linear calibration curves ($r > 0.99$).

However, since EDTA is present in the assay buffer, the actual Ca^{2+} concentration is presumably higher.

"Jump dilution" experiment.

Sample preparation			Assay plate		
**TG2 [ng/ml]**	**SH3 [nM]**	**dilution in dH_2O**	**dilution in assay mix**	**TG2/well [ng/ml]**	**SH3/well [nM]**
rhTG2					
7,936.5 (20x)	1530 (20x MC_{50})	1:9	1:1	396.8 (1x)	76.5 (1x MC_{50})
79,365 (200x)	1530 (20x MC_{50})	1:99	1:1	396.8 (1x)	7.65 (0.1x MC_{50})

Table S1. Preparation of "jump dilution" experiment for inhibition mode determination exemplarily with SH3.

Supplementary Results

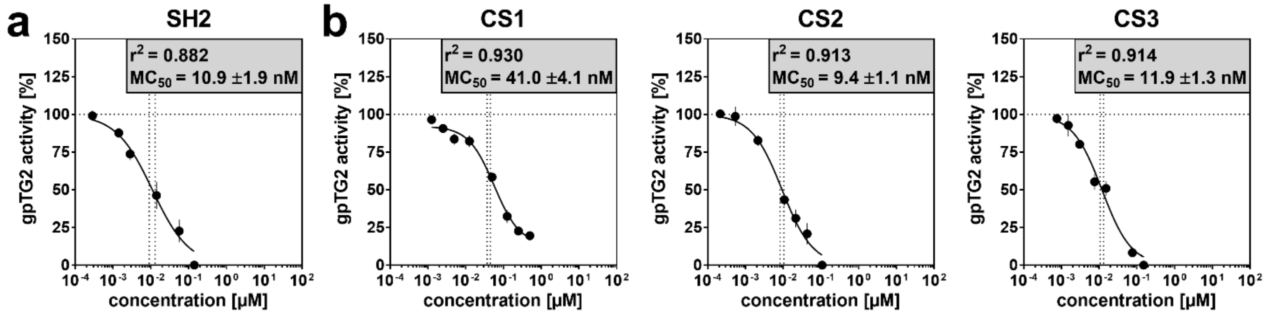

Figure S2. Influence of polymeric GAG derivatives on gpTG2 activity.
gpTG2 were incubated with polymeric GAG derivatives and applied to TG2 activity assay: **a**) SH2, **b**) CS derivatives CS1, CS2 and CS3. Positive control (gpTG2 activity without any treatment) was set to 100%. Values are shown as mean ±SEM; n=3. MC_{50} values were calculated according to non-linear fit (inhibitor concentration vs. activity with variable slope; black line). Vertical dotted lines indicate the range of MC_{50} ±SD.

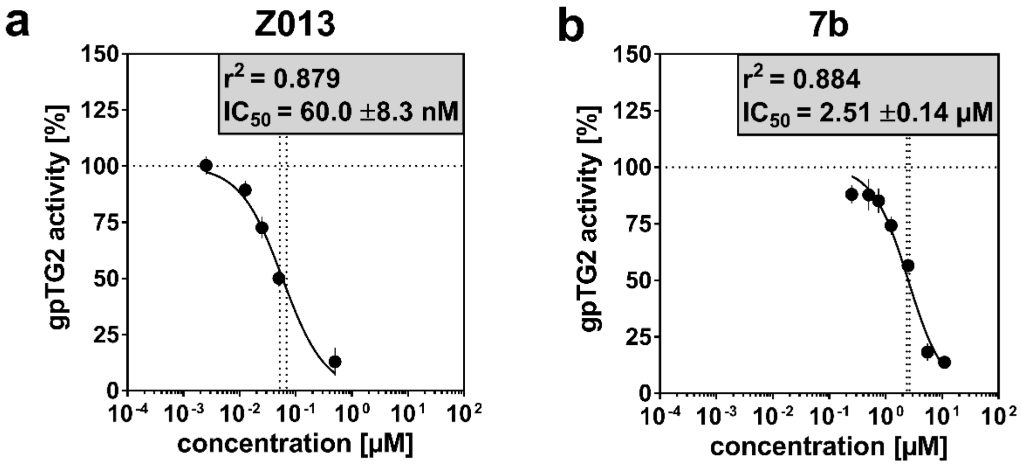

Figure S3. Influence of irreversible inhibitors on gpTG2 activity.
gpTG2 was incubated with inhibitor **a**) Z013 and **b**) 7b, respectively, and applied to TG2 activity assay. Positive control (gpTG2 activity without any treatment) was set to 100%. Values are shown as mean ±SEM; n=3. MC_{50} values were calculated according to non-linear fit (inhibitor concentration vs. activity with variable slope; black line). Vertical dotted lines indicate the range of MC_{50} ±SD.

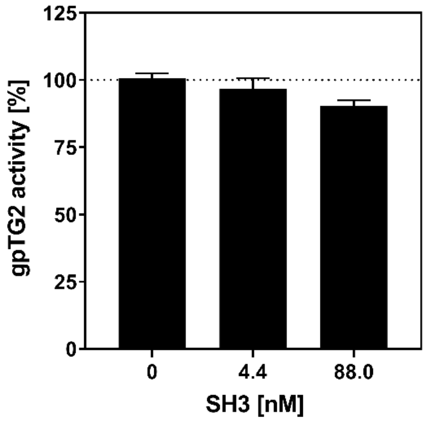

Figure S4. Pre-incubation of the poly-L-lysine coated assay plate with SH3.
Prior to TG2 activity assay, the assay plate (poly-L-lysine coating) was pre-incubated for 5 min at 25°C with H_2O, 4.4 nM and 88 nM SH3, respectively. After briefly washing with H_2O, gpTG2 was mixed with assay buffer and activity assay was performed as described. Positive control (activity of gpTG2 in wells without any pre-incubation) was set to 100%. Values are shown as mean ±SEM; n=3.

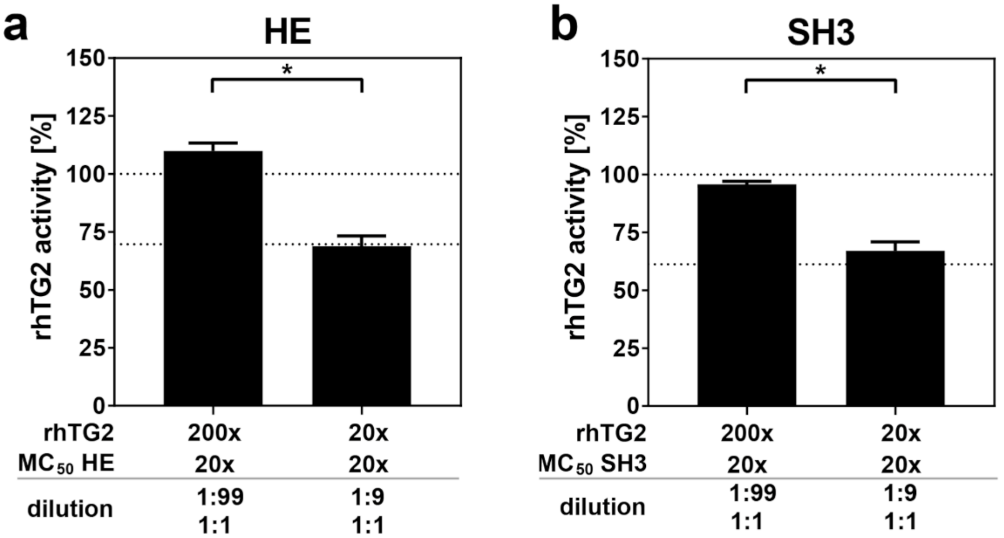

Figure S5. "Jump dilution" experiment with rhTG2 for determination of inhibition mode with HE and SH3.
Higher amounts of gpTG2 (200x or 20x) were incubated with higher amounts of **a**) HE (20x MC_{50}) and **b**) SH3 (20x MC_{50}) for 5 min at 25°C. Before being transferred into the assay plate they were diluted with H_2O either 100-fold or 10-fold. In the assay plate they were further diluted with assay mix 1:1, leading to either $0.1xMC_{50}$ or $1xMC_{50}$ and 1xgpTG2 per well. Values are shown as mean ±SEM; n=3. Significant differences ($p < 0.05$) were analyzed by t-test and are indicated with *. For further details on incubation, see Supplementary Table S1. Horizontal lines indicate positive control (upper line) and expected activity at $1xMC_{50}$ according to inhibition curves (Figure 1).

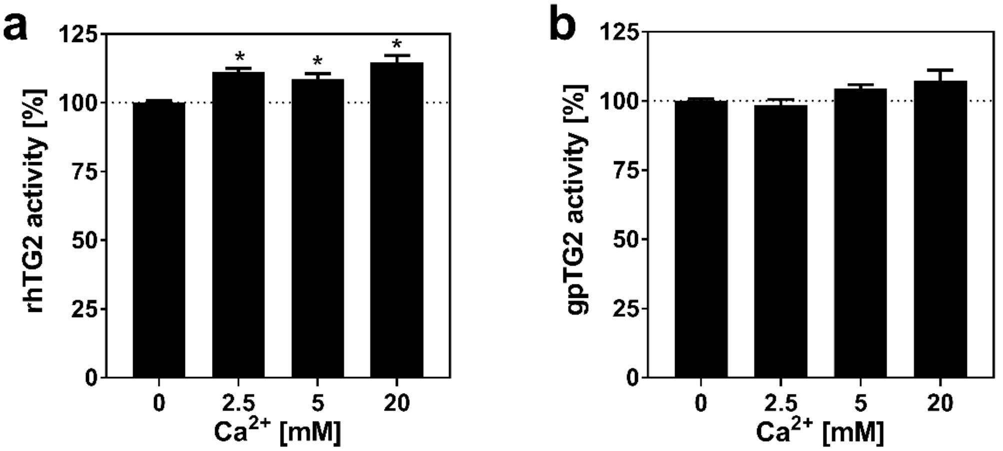

Figure S6. Influence of pre-incubation of TG2 with different Ca^{2+} concentrations on TG2 activity and SH3 inhibitory effect.
Prior to TG2 activity assay, **a**) rhTG2 and **b**) gpTG2 were pre-incubated for 5 min at 25°C with 2.5 mM, 5 mM and 10 mM $CaCl_2$, respectively Afterwards TG2 activity assay was performed as described (Figure 5). Positive control (activity of gpTG2 or rhTG2, respectively, without any pre-incubation) was set to 100%. Values are shown as mean ±SEM; n=3. Significant differences ($p<0.05$) of Ca^{2+} treatment vs. 0 mM $CaCl_2$ were calculated by one-way ANOVA and Bonferroni's post-test and are indicated with *.

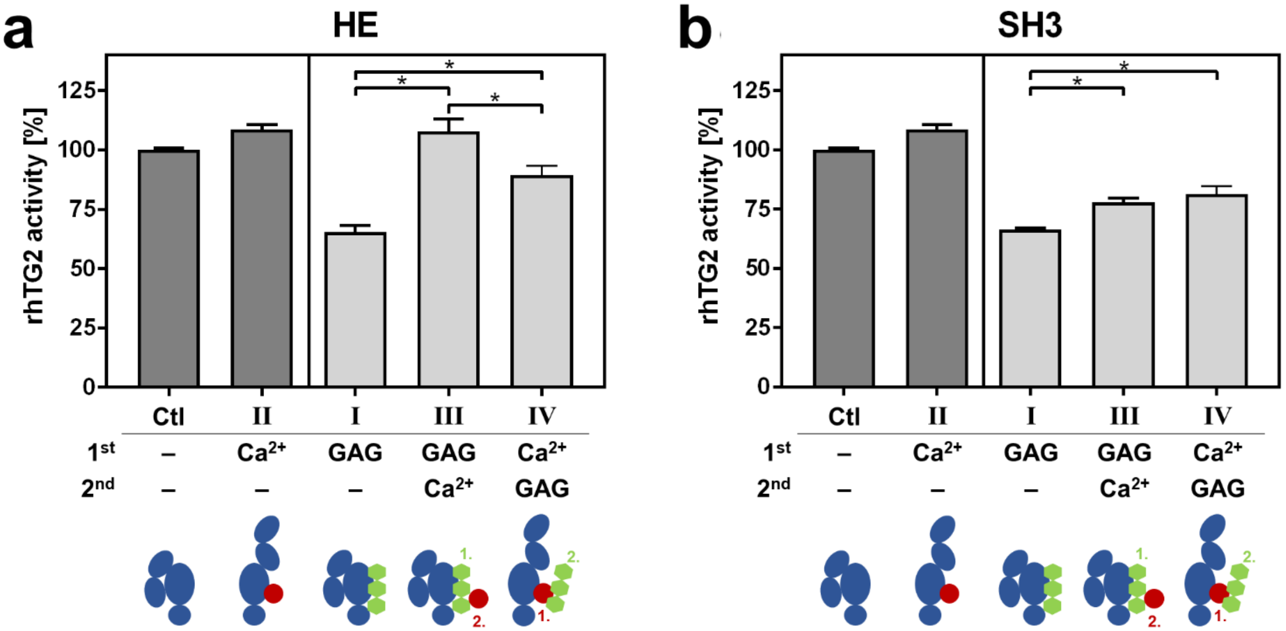

Figure S7. Influence of rhTG2 Ca^{2+} activation on inhibitory effect of sulfated GAG derivatives.
The residual rhTG2 activity was set to around 65% by adding sulfated GAG derivatives. rhTG2 was incubated according to experimental settings I and IV-VI with 5 mM $CaCl_2$ and **a**) HE or **b**) SH3. The given Ca^{2+} concentration refers to that one in the reaction tube before the mixture was applied to the assay plate and assay buffer (with Ca^{2+} in excess) was added. Positive controls (rhTG2 without any treatment "Ctl" and rhTG2 activated with 5 mM $CaCl_2$ concentration "IV") were set to 100%. Values are shown as mean ±SEM; n=3. Significant differences ($p<0.05$) of settings I, V and VI were calculated by one-way ANOVA and Bonferroni's post-test and are indicated with *.

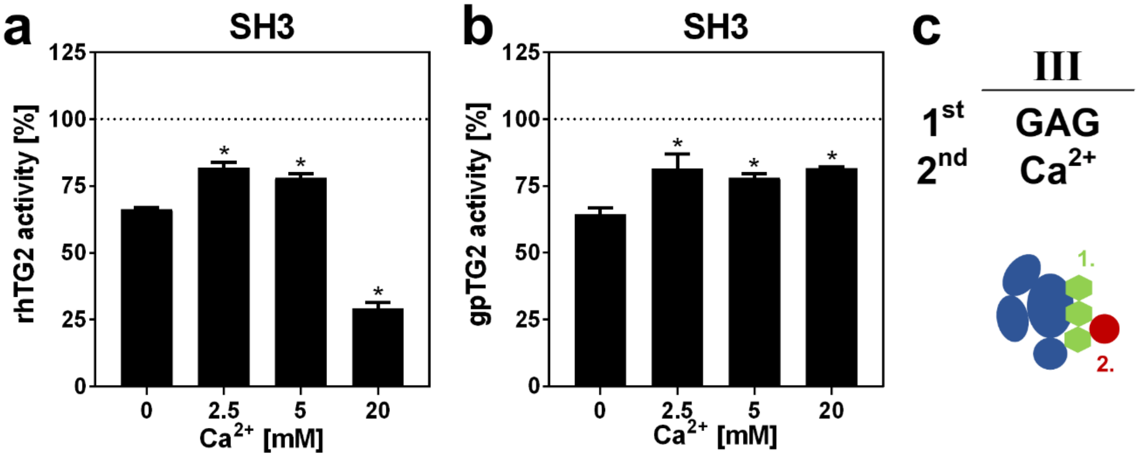

Figure S8. Influence of different Ca^{2+} concentrations on SH3 inhibitory effect.
The residual activity of **a**) rhTG2 and **b**) gpTG2 was set to around 65% by adding SH3. TG2 was further incubated according to **c**) experimental setting V with 2.5, 5 or 20 mM $CaCl_2$. The given Ca^{2+} concentration refers to that one in the reaction tube before the mixture was applied to the assay plate and assay buffer (with Ca^{2+} in excess) was added. Positive controls (TG2 without any treatment) were set to 100%. Values are shown as mean ±SEM; n=3. Significant differences ($p<0.05$) in comparison to 0 mM Ca^{2+} were calculated by one-way ANOVA and Bonferroni's post-test and are indicated with *.

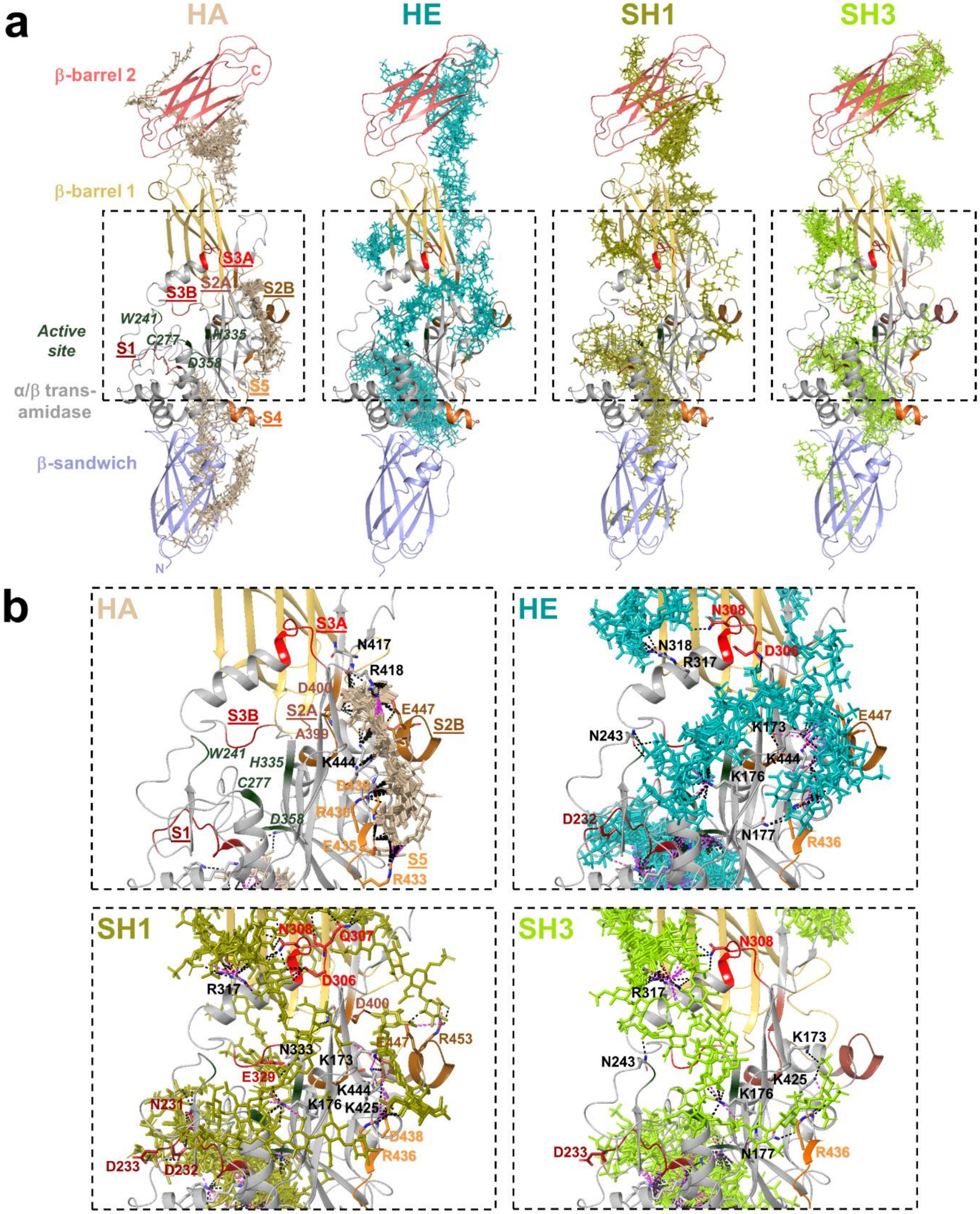

Figure S9. Molecular modeling of the interaction of GAG derivatives with rhTG2 in open conformation.
Docking results obtained using Glide. The four domains of rhTG2 are shown in cartoon: β-sandwich (purple), α/β-transamidase domain (gray), β-barrel 1 (yellow) and β-barrel 2 (salmon). **a**) The different GAG clusters are shown in sticks: HA (pale), HE (teal), SH1 (smudge) and SH3 (green). **b**) Zoom-in of the β-barrel 1 and α/β-transamidase domain region according to the dashed boxes shown in panel **a**. Residues at the active site are highlighted in dark green. Ca^{2+} binding sites are highlighted in dark red (S1), brown (S2), red (S3) and orange (S4, S5). H-bonds (black) and salt bridges (pink) are visualized as dashed lines. Figure generated in *Maestro* (v12.3)[2].

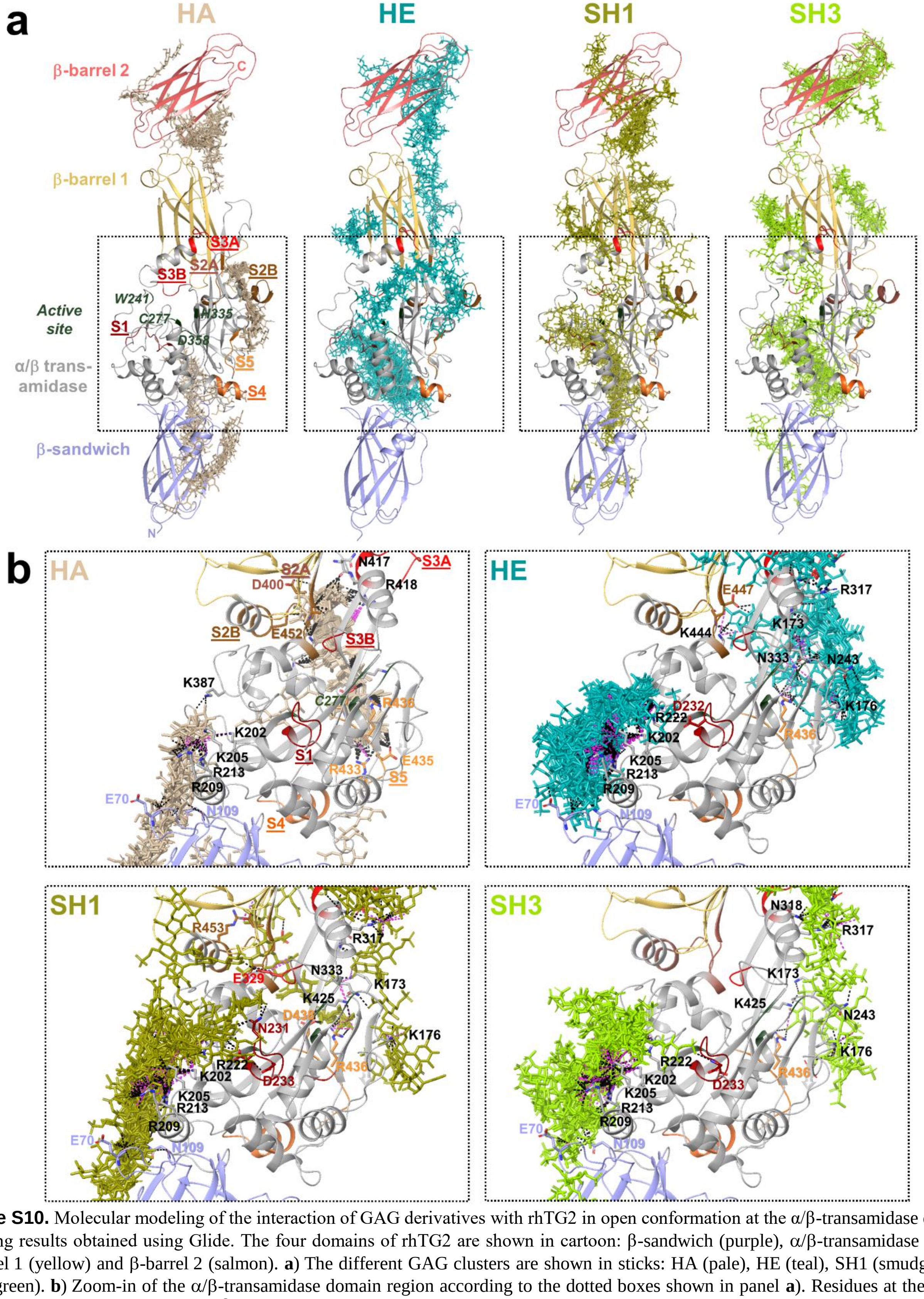

Figure S10. Molecular modeling of the interaction of GAG derivatives with rhTG2 in open conformation at the α/β-transamidase domain. Docking results obtained using Glide. The four domains of rhTG2 are shown in cartoon: β-sandwich (purple), α/β-transamidase (gray), β-barrel 1 (yellow) and β-barrel 2 (salmon). **a**) The different GAG clusters are shown in sticks: HA (pale), HE (teal), SH1 (smudge) and SH3 (green). **b**) Zoom-in of the α/β-transamidase domain region according to the dotted boxes shown in panel **a**). Residues at the active site are highlighted in dark green. Ca^{2+} binding sites are highlighted in dark red (S1), brown (S2), red (S3) and orange (S4, S5). Interacting rhTG2 residues are highlighted in sticks and numbered. H-bonds (black) and salt bridges (pink) are visualized as dashed lines. Figure generated in *Maestro* (v12.3)[2].

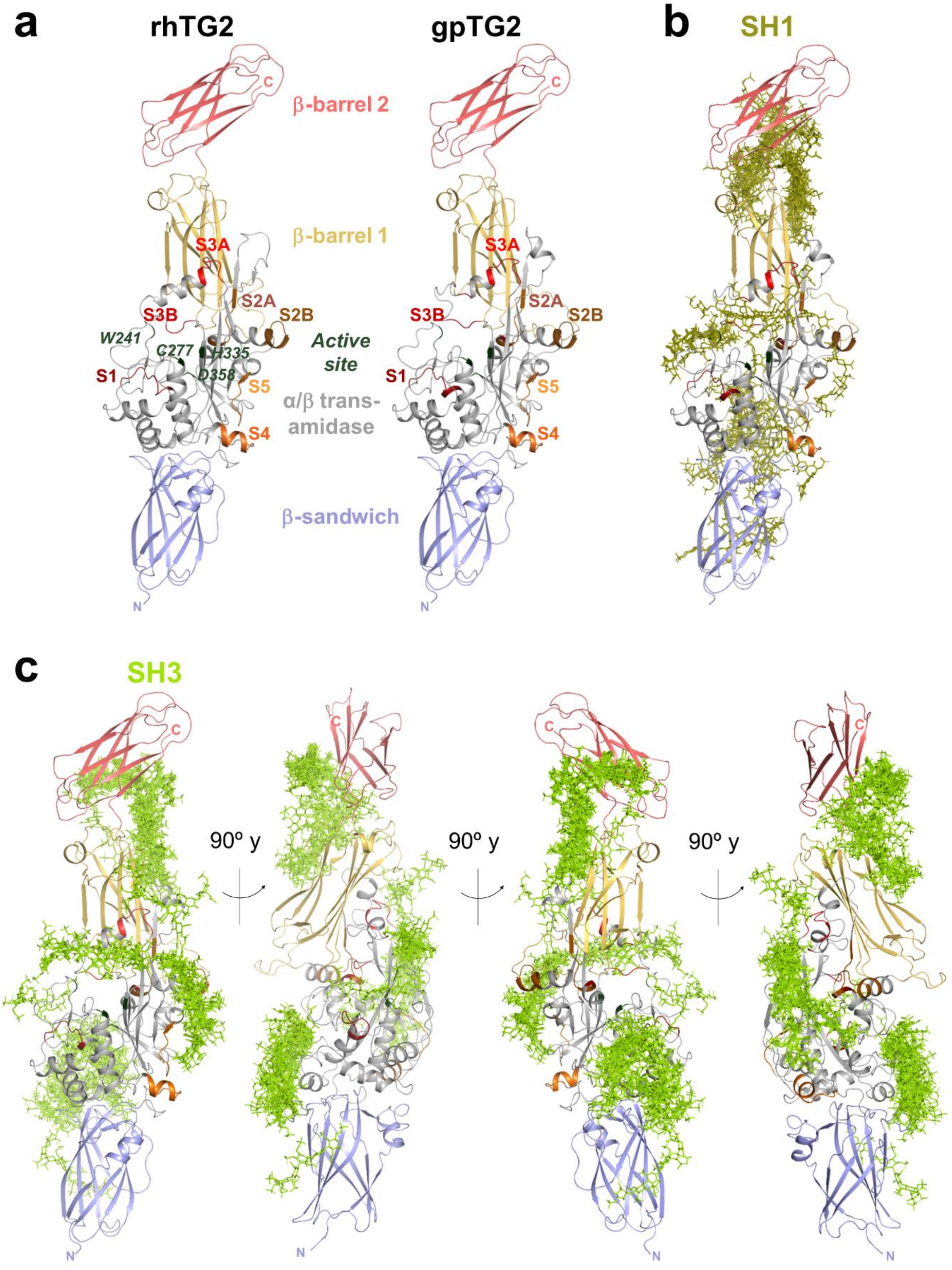

Figure S11. Molecular modeling of GAG recognition by gpTG2 in open conformation.

a) Comparable view of rhTG2 *vs.* gpTG2. The four domains are shown in cartoon: β-sandwich (purple), α/β-transamidase (gray), β-barrel 1 (yellow) and β-barrel 2 (salmon). Residues at the active site are highlighted in dark green. Ca^{2+} binding sites are highlighted in dark red (S1), brown (S2), red (S3) and orange (S4, S5). Docking results obtained using Glide. The different GAG clusters are shown in sticks: **b**) SH1 (smudge) and **c**) SH3 (green). For gpTG2/SH3-complex (**c**) three additional views after 90° stepwise turns y-axis are depicted. Figures generated in *Maestro* (v12.3)[2].

HS/HE binding clusters	Involved TG2 domains	Reference
R19, R28, $_{262}$RRWK$_{265}$, $_{598}$KQKRK$_{602}$, K634	β-sandwich, α/β-transamidase domain, β-barrel 2	[3] [Lortat-Jacob et al., 2012]
$_{202}$KFLKNAGRDCSRRSSPVYVGR$_{222}$	α/β-transamidase domain	[4] [Wang et al., 2012]
$_{202}$KFLKNAGRDCSRRS$_{215}$, $_{261}$LRRWKNHGCQRVKY$_{274}$	α/β-transamidase domain	[5] [Teesalu et al., 2012]

Table S2. Previously identified HS/HE binding clusters on rhTG2.

**β-sandwich[a,b] (1-139)**		**α/β-transamidase[a,c] (147-460)**			**β-barrel 1[a] (472-583)**		**β-barrel 2[b] (591-690)**
SH3							
Y50	R30	R240 (**cat**)	Q238	N318 (**cat**)	R481	V526	T626
R76		N244	R240	N326 (**cat**)	Q484	K553	K634
S78		Y245	R263	K327	T487	R567	Q636
S80		S253	Q270	K408			S638
S81		R262	R271	-------------------			N670
S129		R263	K273	L312			K675
		K265		K414			K677

Table S3: Predicted recognition sites of gpTG2 in closed conformation by SH3 hexasaccharide.

[a] Different recognition sites are shown in different columns or in the same column separated by dashed lines. [b] Underlined residues correspond to low populated GAG recognition sites. [c] It includes catalytic core (triad C277, H335, D358) and Ca^{2+} binding sites (**S1** ($_{226}$AMVNCNDD$_{233}$), **S2A** ($_{396}$EVNADV$_{401}$), **S2B** ($_{446}$EGSEEERE$_{453}$), **S3A** ($_{306}$DQNSNLL$_{312}$), **S3B** ($_{328}$SEM$_{330}$), **S4** ($_{151}$DSDQERQE$_{158}$) and **S5** ($_{432}$RDERED$_{437}$)).

**β-sandwich**[a] **(1-139)**		**α/β-transamidase**[a,b,c] **(147-460)**			**β-barrel 1**[a,d] **(472-583)**		**β-barrel 2**[a,d,e] **(591-687)**	
HA								
E13	Q69	K202	A399 (**S2A**)		N484		*E588*	*K590*
D20	E70	K205	D400 (**S2A**)		R478		R592	Q610
Q37	K74	R209	N417		R580		E669	E646
D94	R76	R213	R418		*E585*		K677	K649
Q103	R80	K387	K425				R680	
	E85		R433 (**S5**)				N681	
	D87		E435 (**S5**)					
	N109		R436 (**S5**)					
			D438 (**S5**)					
			K444					
			E447 (**S2B**)					
			E452 (**S2B**)					
HE								
	R35	K202	K173 (**cat**)	N308 (**S3A**)	R476	R512	*K590*	K598
	E70	K205	K176 (**cat**)	R317 (**cat**)	R478	K527	R592	K677
	N109	N206	N177	N318	K540	N531	K649	R680
		R209	N243	N333 (**cat**)	R580	R564	R651	N681
		R213	A304 (**cat**)					
		R222	D306 (**S3A**)					
		D232 (**S1**)	N333 (**cat**)					
		K387	R418					
			K425					
			R436 (**S5**)					
			K444					
			E447 (**S2B**)					
SH1								
S56	Q69	K202	K173 (**cat**)	A304 (**cat**)	R476	R512	*N586*	K634
R80	E70	K205	K176 (**cat**)	D306 (**S3A**)	R478	E523	*E588*	K663
S122	K74	R209	N398 (**S2A**)	Q307 (**S3A**)	T496	K527	*K590*	E669
	R76	R213	D400 (**S2A**)	N308 (**S3A**)	E539		R592	K677
	D87	R222	K425	R317 (**cat**)	K540		K649	
	N109	N231 (**S1**)	R436 (**S5**)	N318	R580		R651	
		D232 (**S1**)	D438 (**S5**)	N333 (**cat**)			K674	
		D233 (**S1**)	K444				K677	
		E329 (**S3B**)	E447 (**S2B**)				R680	
		K387	R453 (**S2B**)				N681	
SH3								
S56	Q69	K202	K173 (**cat**)	N243 (**cat**)	R476	R512	*K590*	
Q69	E70	K205	K176 (**cat**)	N308 (**S3A**)	R478	E523	R592	
R76	N109	R209	N177	R317 (**cat**)	N498	K527	K598	
R116		R213	K425	N318	R580	K550	K649	
		R222	R436 (**S5**)				R651	
		D233 (**S1**)					R680	
		K387					N681	

Table S4. Predicted recognition sites for GAG hexasaccharides on rhTG2 in open conformation.

[a] Different recognition sites are shown in different columns. [b] D408 is recognized by all (sulfated) GAG distributed at the β-barrel 1 site (left column). [c] It includes catalytic core (**cat**) (triad C277, H335, D358) and Ca^{2+} binding sites (**S1** ($_{226}$GMVNCNDD$_{233}$), **S2A** ($_{396}$EVNADV$_{401}$), **S2B** ($_{447}$EGSSEERE$_{454}$), **S3A** ($_{306}$DQNSNLL$_{312}$), **S3B** ($_{328}$SEM$_{330}$), **S4** ($_{151}$DSEEERQE$_{158}$) and **S5** ($_{433}$RDERED$_{438}$)). [d] Residues between contiguous domains are shown in italic. [e] A very low populated cluster involving residues D640, R651 and K634 has been predicted for HA.

β-sandwich[a,b] (1-139)		α/β-transamidase[a,c] (147-460)			β-barrel 1 (472-583)	β-barrel 2[d] (592-690)
SH1						
T56	T87	K202	K424	K173 (**cat**)	R479	*N589*
R76	E69	Q209	R435 (**S5**)	R240 (**cat**)	R481	E591
S78	E70	R213		N243	N485	K593
E84		R216		R317 (**cat**)	R583	R595
E120		R222		N318 (**cat**)		K613
Q126		K387		N326 (**cat**)		Q650
				K327		K652
				Q362		R683
				K364		
				R406[e]		
				Q407[e]		
SH3						
N60	D66	K202	K380	K173 (**cat**)	R479	*N589*
R76	E69	K205	K424	R240 (**cat**)	R481	E591
R116	E70	N206	R435 (**S5**)	N243	N485	K593
		Q209	D437 (**S5**)	N244	R583	R595
		R213		N302		K613
		R216		S303 (**cat**)		Q650
		R222		A304 (**cat**)		K652
		K387		Q307 (**S3A**)		K666
				N310 (**S3A**)		R683
				R317 (**cat**)		N684
				N318 (**cat**)		
				N326 (**cat**)		
				N333 (**cat**)		
				R406[e]		
				Q407[e]		
				K414		

Table S5. Predicted recognition sites of gpTG2 in open conformation by SH1 and SH3 hexasaccharides.
[a] Different recognition sites are shown in different columns or in the same column separated by dashed lines. [b] Underlined residues correspond to low populated GAG recognition sites. [c] It includes catalytic core (triad C277, H335, D358) and Ca^{2+} binding sites (**S1** ($_{226}$AMVNCNDD$_{233}$), **S2A** ($_{396}$EVNADV$_{401}$), **S2B** ($_{446}$EGSEEERE$_{453}$), **S3A** ($_{306}$DQNSNLL$_{312}$), **S3B** ($_{328}$SEM$_{330}$), **S4** ($_{151}$DSDQERQE$_{158}$) and **S5** ($_{432}$RDERED$_{437}$)). [d] Residues between contiguous domains are shown in italic. [e] Bridging recognition residues between the α/β-transamidase and β-barrel 1 domains.

Supplementary Discussion

Open TG2 also reveals manifold molecular recognition sites for GAG.

Previous experimental data suggested an interaction of GAG with TG2 not only in closed conformation but in open conformation as well, although with a weaker binding affinity[4]. Therefore, further docking studies with the enzyme's open conformation were performed in four steps involving each TG2 domain (*i.e.* β-sandwich, α/β-transamidase, β-barrel 1, β-barrel 2). In addition, a partial overlap of contiguous domains to the main protein domain considered for docking was applied (see Materials and Methods). As before, GAG hexasaccharides were predicted to bind along the four domains of both orthologous enzymes in open conformation (Supplementary Figures S9-S10 and Supplementary Table S4 for rhTG2, Supplementary Figure S11 and Supplementary Table S5 for gpTG2).

For rhTG2 β-barrel 2, all investigated GAG derivatives (*i.e.* HA, HE, SH1 and SH3) were predicted to interact with residues K590, R592, K649, R651, R680 and N681. In the case of HA and SH1, additional binding poses were observed involving residue K634, which has been previously reported to be involved in HS/HE recognition by TG2 in closed conformation[3]. All investigated GAG derivatives were disposed between the β-barrel 2 and β-barrel 1 domains through interactions with R478

and R580 (Supplementary Figure S9, Supplementary Table S4). Further, the GAG derivatives also showed additional interactions with D408 at the α/β-transamidase domain surrounding the β-barrel 1.

The results obtained from the GAG docking to the α/β-transamidase domain of rhTG2 are visualized in detail in Supplementary Figure S9B. GAG recognized the reported Ca^{2+} binding site S5[6] to a different extent in which HA was showing the maximum number of interactions (Supplementary Table S4). Binding poses for HA and SH1 were distributed along S2A and showed contacts with N398, A399 and D400. Further, HA, HE and SH1 were shown to recognize residue E447 at S2B. As a common theme for HE, SH1 and SH3, binding poses along the catalytic core comprising residues from S3A were obtained. In addition, they showed interactions with K173, K176, R317 and N333 (the last-named is not observed with SH3) being more populated for HE and SH3 than for SH1. On the other hand, only SH1 showed contacts with E329 at S3B, although low populated. All investigated sulfated GAG derivatives (HE, SH1, SH3) occupied S1 and exhibited contacts with either N231, D232 and/or D233 (Supplementary Figure S9b, Supplementary Table S4).

The results of the molecular docking calculations revealed that the investigated GAG derivatives participated in interactions with protein residues K202, K205, R213, R222 and K387 (Supplementary Figure S10, Supplementary Table S4) resembling binding sites described for HS/HE (see Supplementary Table S2)[4,5]. This recognition site served simultaneously as path to further bridge the α/β-transamidase and β-sandwich domains through additional contacts with N109 and the backbone of E70 (Supplementary Figure S10). At the β-sandwich domain, sulfated GAG derivatives showed low populated clusters having interactions with Q69 and R76, while R116 showed H-bonds with sulfate groups of SH3 (see Supplementary Table S4). Noteworthy, R116 has been described as a critical residue for fibronectin recognition[7,8]. In the case of HA, some poses were also shown in which contacts are established through hydroxyl groups with the side chain of residues E85 and D94, which are part of another reported binding site for fibronectin[9] and the TG2 inhibitor GK921[10].

Molecular docking studies with SH1 and SH3 hexasaccharides and gpTG2 in the open conformation were performed as for the human enzyme (Supplementary Figure S11b,c). A summary of gpTG2 residues involved in sulfated GAG recognition is given in Supplementary Table S5. SH1 and SH3 were predicted to be distributed along the β-barrel 2 and making contacts with residues E591, K593, R595, K613, Q650, K652, and R683. These interactions resemble binding sites of the human enzyme isoform (the rhTG2/gpTG2 residue correspondences are as follows: R592/R595 and R680/R683). As for rhTG2, SH1 and SH3 were found bridging both β-barrel domains in gpTG2 through interaction with residues R479, R481, R583, N485 and N589. Furthermore, both sulfated GAG derivatives showed additional interactions with the nearby α/β-transamidase residues R406 and Q407. As previously observed for rhTG2, binding poses of sulfated GAG derivatives were also predicted at the α/β-transamidase domain involving as well the Ca^{2+} binding sites S3 and S5, with the obtained clusters being more populated for SH3 than for SH1, and also more than in rhTG2 (Figure 3d, Supplementary Figure S11b,c, Supplementary Table S5). Moreover, an additional highly populated cluster described by interactions with residues K202, Q209, R216, R222 and K387 was predicted. Binding poses occupying the Ca^{2+} binding site S1 were less populated than those predicted for rhTG2. Like for rhTG2, the two investigated sulfated GAG derivatives were also found to bind between the α/β-transamidase and β-sandwich domains using a similar interacting path comprised by residues K387, R216, R213 and E70 for one cluster and Q209, N206, K202, K205 (only for SH3) and D66 for a second cluster. Poses at the β-sandwich domain were also predicted for both sulfated GAG derivatives, although less populated than in rhTG2 in the case of SH3. SH1 was found to interact with T56, S78, R76 and E84, resembling the predicted site for the recombinant human orthologue.

The predicted binding clusters of hexasaccharidic GAG to rh/gpTG2 presume that polymeric GAG chains might, on one side, stabilize the TG2 closed conformation, but also the TG2 open form, while competing for Ca^{2+} binding sites. Supplementary Figure S11c shows molecular details of the gpTG2/SH3 complex illustrating the SH3 disposition along the surface of the enzyme.

Overall, the presented theoretical models predicted that only sulfated GAG derivatives (HE, SH1, and SH3), in contrast to the non-sulfated HA, could occupy a region comprising exposed residues from the TG2 catalytic site and, thus, could affect TG2 activity. In particular, this would allow the conjecture, that sulfated GAG derivatives might prevent substrate binding by steric hindrance, as the substrates (e.g. fibronectin) are rather large. However, experimental results rule out an interference of sulfated GAG with substrate binding in open conformation, since activity of Ca^{2+}-activated TG2 was not impaired.

With respect to the possible interaction of GAG with different Ca^{2+} binding sites, all investigated GAG were able to recognize the rhTG2 S5 Ca^{2+} binding site. S1 was recognized by all sulfated GAG derivatives, and S2 (A and B) by HA and SH1, S2B also by HE. The S3A Ca^{2+} binding site showed interactions with all studied sulfated GAG derivatives (Supplementary Table S4). In case of gpTG2, a similar scenario was predicted with SH1 and SH3 interacting with residues of S3A and S5 (Supplementary Table S5). Interestingly, docking results indicated that SH3 was the most populated sulfated GAG derivative in such Ca^{2+} binding sites for gpTG2 (Supplementary Figure S9, S11). According to mutagenesis studies by Király et al.[6], the S3 Ca^{2+} binding site was reported to be essential for TG2 activity as it is required to open the active site and the substrate channel. The S1 Ca^{2+} binding site, although representing a strong recognition site, is not determinant for TG2 activity. While S2 has a minor role in TG2 activity, it can have a cooperative role with other Ca^{2+} binding sites. Similarly, it has been proposed that S4 and S5 possess a cooperative role with S3[6]. The molecular docking results in open conformation revealed that sulfated GAG derivatives might compete with Ca^{2+} for binding to the critical S3 binding site and thus reduce TG2 activity.

Supplementary References

1. Hempel, U. *et al.* Artificial Matrices With High-Sulfated Glycosaminoglycans and Collagen Are Anti-Inflammatory and Pro-Osteogenic for Human Mesenchymal Stromal Cells. *J. Cell Biochem.* **115**, 1561–1571, DOI: 10.1002/jcb.24814 (2014).
2. *Schrödinger Release 2019-1/4: Maestro LLC*. (Schrödinger, Inc., 2019).
3. Lortat-Jacob, H. *et al.* Transglutaminase-2 Interaction with Heparin: Identification of a Heparin Binding Site that Regulates Cell Adhesion to Fibronectin-Transglutaminase-2 Matrix. *J. Biol. Chem.* **287**, 18005–18017, DOI: 10.1074/jbc.M111.337089 (2012).
4. Wang, Z. *et al.* Characterization of Heparin-binding Site of Tissue Transglutaminase: Its Importance in Cell Surface Targeting, Matrix Deposition, and Cell Signaling. *J. Biol. Chem.* **287**, 13063–13083, DOI: 10.1074/jbc.M111.294819 (2012).
5. Teesalu, K., Uibo, O., Uibo, R. & Utt, M. Kinetic and functional characterisation of the heparin-binding peptides from human transglutaminase 2. *J. Pept. Sci.* **18**, 350–356, DOI: https://doi.org/10.1002/psc.2413 (2012).
6. Király, R. *et al.* Functional significance of five noncanonical Ca2+-binding sites of human transglutaminase 2 characterized by site-directed mutagenesis. *FEBS J.* **276**, 7083–7096, DOI: https://doi.org/10.1111/j.1742-4658.2009.07420.x (2009).
7. Cardoso, I. *et al.* Transglutaminase 2 interactions with extracellular matrix proteins as probed with celiac disease autoantibodies. *FEBS J.* **282**, 2063–2075, DOI: 10.1111/febs.13276 (2015).
8. Cardoso, I. *et al.* Dissecting the interaction between transglutaminase 2 and fibronectin. *Amino Acids* **49**, 489–500, DOI: 10.1007/s00726-016-2296-y (2017).
9. Hang, J., Zemskov, E. A., Lorand, L. & Belkin, A. M. Identification of a Novel Recognition Sequence for Fibronectin within the NH2-terminal β-Sandwich Domain of Tissue Transglutaminase. *J. Biol. Chem.* **280**, 23675–23683, DOI: 10.1074/jbc.M503323200 (2005).
10. Kim, N. *et al.* Allosteric inhibition site of transglutaminase 2 is unveiled in the N terminus. *Amino Acids* **50**, 1583–1594, DOI: 10.1007/s00726-018-2635-2 (2018).
